# Recent advances in training intensity distribution theory for cyclic endurance sports: theoretical foundations, model comparisons, and periodization characteristics

**DOI:** 10.3389/fphys.2025.1657892

**Published:** 2025-10-15

**Authors:** Qihao Sun, Yin Yu, Jiayue Cui, Simin Lin, Xiaohan Wang, Tian Zhou

**Affiliations:** 1 School of Sports Training, Wuhan Sports University, Wuhan, China; 2 Research Center for High-Quality Development of Characteristic Competitive Sports, Wuhan Sports University, Wuhan, China

**Keywords:** cyclic endurance sports, training model, training load, training intensity distribution, endurance training

## Abstract

Training Intensity Distribution (TID) has emerged as a crucial component of training load regulation, providing a critical foundation for designing endurance training programs and optimizing performance. This paper systematically reviews the theoretical underpinnings of TID and the evolution of training intensity zone classification. The distribution characteristics, underlying physiological mechanisms, and contextual applicability of three representative models—pyramidal (PYR), threshold (THR), and polarized (POL)—are examined in particular. The functional roles of each TID model across different endurance disciplines and training scenarios are further examined in this study, which draws on recent empirical evidence. It focuses on their dynamic structural shifts throughout the general preparation, specific preparation, pre-competition, and competition phases. The findings demonstrate that each TID model exhibits distinct structural and application scenarios and can significantly improve performance development when aligned with the specific demands of training phases and sport-specific tasks. It should be determined based on sport characteristics, training objectives, and individual variability when choosing an appropriate TID model in practice. Additionally, phased adaptations and strategic integration of multiple TID models may enhance cumulative training adaptations and competitive performance. This approach provides theoretical support for the scientific management of endurance training in cyclic sports.

## 1 Introduction

As a dynamic process of adaptive development, athletic training gradually improves athletic performance through scientifically formulated methodologies and interventions to achieve optimal competitive results (Yu and Hu, 2019). Coaches stimulate the athlete’s body systematically by scientifically arranging training loads and investigate the individual’s physiological and functional adaptations to training via comprehensive monitoring and assessment (Ma and Wu, 2022). This method progressively uncovers the “black box” mechanisms that govern the development and evolution of competitive ability (Li, 2015).

Training Intensity Distribution (TID) is known as the proportion of training duration across different intensity levels at a specified training phase (such as annual plans, macrocycles, or mesocycles). This is a fundamental metric for regulating the training load of cyclic endurance sports, suitable for events such as middle- and long-distance running, cross-country skiing, road cycling, rowing, canoeing, and distance swimming. TID stands for the strategic approach to training load management, which is crucial for striking a balance between physiological adaptation, performance results, and injury risk. A growing body of research indicates that well-structured TID models strategically modulate the training intensity, duration, and frequency, which is essential for optimizing athletic capacity, improving performance, and attaining exceptional results (Seiler, 2010).

Cyclic endurance events, a fundamental component of professional sports, rely heavily on planned training load distribution to promote adaptation and performance. Despite being predominantly aerobic, these sports differ greatly in terms of energy requirements, technical execution, and competitive formats. Meanwhile, these disparities result in a great deal of variation in intensity zone classification, training load distribution tactics, and periodization models across several fields. The core problem is how to accurately calibrate the distribution of intensity zones throughout training phases (like general preparation, specific preparation, pre-competition, and competition) so as to guarantee progressive training objectives and the enhancement of physiological adaptations (Hu and Jin, 2020; Ma et al., 2025). This paper seeks to provide a comprehensive analysis of the theoretical underpinnings, historical development, and exemplary models of TID. Additionally, it examines the structural dynamics and functional roles of TID within the training cycle, providing theoretical and practical guidance for the optimization and scientific management of training in cyclic endurance sports.

Although this review is narrative in nature rather than a systematic review, a structured search strategy was employed to ensure transparency in the literature selection process. Relevant publications were identified through systematic searches of major academic databases including PubMed, Web of Science, Scopus, and CNKI. Key search terms included “training intensity distribution,” “endurance training,” “pyramidal model,” “threshold training,” and “polarized model.” We included both foundational and recent studies (1979–2025) to capture the historical evolution and contemporary advances in exercise physiology. Additional relevant references were identified by screening the reference lists of key review articles. A total of ten relevant publications were included in this review.

Inclusion criteria comprised peer-reviewed studies that provided empirical data or theoretical analysis on TID models in endurance athletes or related training interventions. Non-peer-reviewed sources, case studies with insufficient methodological detail, and studies unrelated to endurance training were excluded. To enhance the transparency and reproducibility of this narrative review, we have included a summary table of the core studies that form the basis of our discussion (see Table 1). This table provides key characteristics of these studies, including their sport, sample, and findings.

**TABLE 1:** Summary of key characteristics of included studies.

Study	Sport	Sample characteristics	Measurement methods	TID model	Key findings
Kindermann et al. (1979)	Not mentiononed	n = 20	Blood lactate, ventilatory thresholds	THR (conceptual origin)	Foundation for THR model
Billat et al. (2001)	Running (marathon)	Elite runners	HR, lactate, velocity	PYR	Typical PYR distribution observed; improved vVO_2_max and running economy
Seiler and Kjerland (2006)	Cross-country skiing	n = 12, world-class athletes	HR, RPE, lactate	POL	Introduced POL with 75%–80% Z1, 5% Z2, 15%–20% Z3
Zapico et al. (2007)	Cycling	n = 14	HR, lactate	PYR	Distance-based training load showed PYR; session-based metrics suggested POL
Guellich et al. (2009)	Rowing	n = 15	HR, lactate	PYR → POL	Training shifted from PYR to POL before major competitions
Neal et al. (2013)	Cycling	n = 22	HR, lactate, MLSS	POL vs. THR	POL produced greater VO_2_max and TT improvements
Stöggl and Sperlich (2014)	Running & Cycling	n = 48	HR, VO_2_, lactate	POL vs. THR vs. HIIT	POL showed superior VO_2_max and performance gains compared with THR and HIIT
Pérez et al. (2020)	Ruuning	n = 20, ultra-endurance runners	VO_2_max, fat metabolism analysis, MVC & RFD	POL vs. THR	POL improved VO_2_max and fat metabolism; THR better maintained neuromuscular function
Filipas et al. (2022)	Running (5 km)	n = 60	HR, VO_2_, TT performance	PYR → POL	Sequential PYR-to-POL led to greatest VO_2_max and 5 km TT gains (+1.5%)
Sperlich et al. (2023)	Triathlon	n = 30	HR, VO_2_, lactate	Mixed PYR/POL	Highlighted model integration depending on training phase and event demands

*POL*, polarized training; *PYR*, pyramidal training; *THR*, threshold training; *HIIT*, high intensity interval training; *HR*, heart rate; RPE, rating of perceived exertion in the Borg 6–20 scale; *VO*_*2max*_, maximum oxygen uptake;*vVO*_*2max*_, velocity of maximum oxygen uptake; *MLSS*, maximum lactate steady state; *TT*, time trail; *MVC*, maximal voluntary contraction; *RFD*, rate of force development.

Nevertheless, it is important to acknowledge the limitations of this approach. Unlike a systematic review, our narrative method did not employ a pre-registered protocol, a formal risk of bias assessment, or a quantitative synthesis of findings. This could introduce selection bias and limit the overall reproducibility. Accordingly, the findings presented in this review should be interpreted as an integrative synthesis of current knowledge rather than a definitive systematic evaluation.

## 2 Theoretical foundations and evolutionary trajectory

Training Intensity Distribution (TID) refers to the training time proportion allocated to different intensity levels during regular training. It creates a systematic training load framework by combining training load intensity and training duration (Seiler and Tønnessen, 2009). TID, which is a significant research direction in sports science, has become a focus with the systematic advances in foundational studies concerning the classification, structural characteristics, physiological effects, and training load monitoring. In-depth research on this theme has broadened the theoretical dimensions of training load and provided new scientific evidence to back up the refined regulation of training practices. TID is critical in training program design and endurance performance optimization (Stöggl and Sperlich, 2015).

### 2.1 Origins and theoretical basis of training intensity distribution

Training Intensity Distribution (TID), which is the training duration proportion assigned to various intensity levels throughout standard training regimens, integrates training load intensity and duration into a systematic training load framework (Seiler and Tønnessen, 2009). Talent Identification and Development (TID) has emerged as a prominent research focus in sports science after systematic advancements in foundation research on classification, structural characteristics, physiological consequences, and training load monitoring. This study has broadened the training load theory and yielded new scientific data to enhance the regulation of training practices. Training regimens and endurance performance improvement depend on TID (Stöggl and Sperlich, 2015; Kong et al., 2024).

**FIGURE 1:**
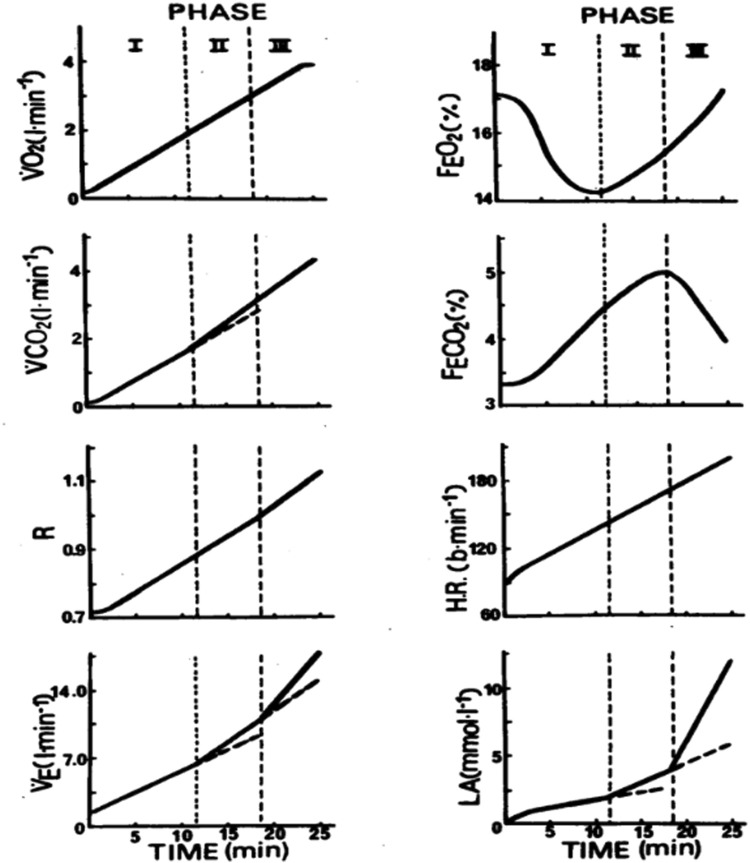
Hemodynamic representation of typical changes in blood lactate, heart rate, and selected gas exchange parameters during progressive exercise from rest to maximal oxygen consumption. PHASE I - PHASE III refers to low to maximal intensity exercise. $\dot{V}$ *O*_*2*_, oxygen uptake per minute; $\dot{\;V}$ *CO*_*2*_, carbon dioxide expired per minute; *R*, respiratory quotient; $\dot{\;V}$ *E*, pulmonary ventilation per minute; *FEO*_*2*_, expired oxygen concentration; *FECO*_*2*_, expired carbon dioxide concentration; *HR*, heart rate; *LA*, blood lactate concentration.

The Training Intensity Distribution (TID) theory originated from a profound analysis of the energy metabolism processes during physical activity. ​Skinner’s 1980 research on aerobic-anaerobic transition​ provided the initial conceptual basis, categorizing incremental exercise into low, moderate, and maximal intensity phases. ​This work underscored​ TID’s foundational principles. The research revealed that physiological metrics such as oxygen absorption (VO_2_), carbon dioxide output (VCO_2_), respiratory exchange ratio (RER), ventilation, and heart rate increased linearly with increasing exercise intensity (Figure 1). The nervous system activates type IIa and IIb muscle fibers during the second and third stages, increasing glycolytic capacity and accelerating ATP production. Skinner developed a theoretical model of the aerobic-to-anaerobic transition with these data and summarized its principal characteristics (Table 2).

**TABLE 2:** Hypothetical model of various thresholds and phases selected characteristics during progressive exercise from rest to maximal oxygen consumption.

Phase I	Phase II	Phase III
Aerobic threshold	Anaerobic threshold	
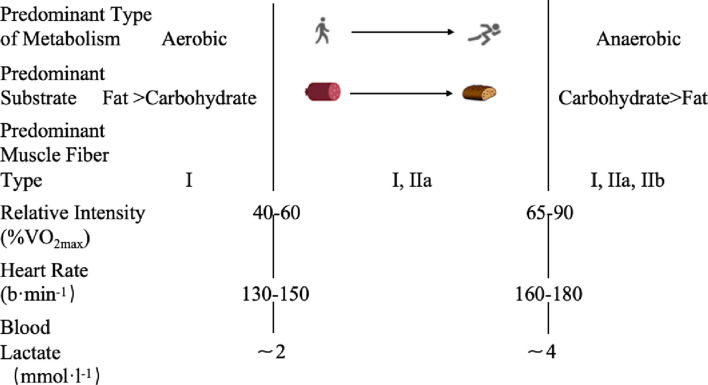		

**FIGURE 2:**
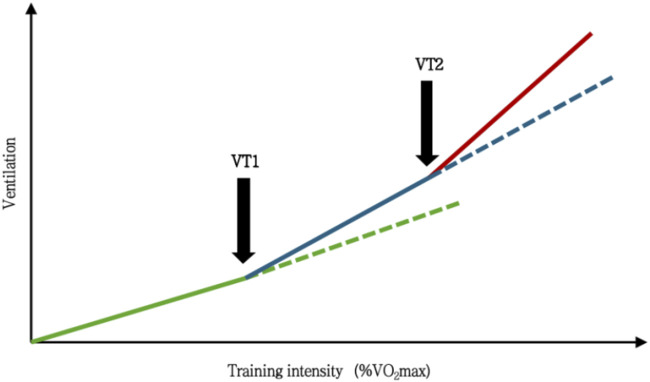
Ventilation Volume-Intensity Curve. *VT1*, first ventilation threshold; *VT2*, second ventilation threshold.

The “Lactate Shuttle Theory” (Brooks, 1986) proposed two pivotal inflection points in lactate metabolism as exercise intensity escalates: the first lactate threshold (LT1) linked to 2 mmol/L blood lactate concentrations and the second lactate threshold (LT2) associated with 4 mmol/L. These thresholds have been extensively utilized as physiological indicators for delineating training intensity zones. The principal energy source in LT1 transitions from fat oxidation to glycolysis with a nonlinear increase in ventilatory rate (V̇E), signifying the first ventilatory threshold (Figure 2). However, LT2 reflects the preponderance of anaerobic metabolism and is typically linked to the second ventilatory threshold (Kong et al., 2024; Pallarés et al., 2016).

### 2.2 The evolution of training intensity zone classification

In the initial phases, numerous scholars established training intensity levels for endurance sports grounded in the previously described energy metabolism features and the theoretical constructs of aerobic and anaerobic thresholds. Endurance training intensity is commonly categorized into three principal zones: low-intensity training (LIT), moderate-intensity training (MIT), and high-intensity training (HIT). Among these, LIT generally refers to exercise performed below the first ventilatory threshold (VT1), MIT spans the range between VT1 and the second ventilatory threshold (VT2), and HIT involves efforts exceeding VT2 (Faude et al., 2009; Wei et al., 2024).

Researchers proposed more sophisticated classification systems as a result of recent study developments, including four-zone and five-zone models predicated on the correlation between training duration and load intensity (Kong et al., 2024). Recent years have witnessed a deepening examination of this topic, resulting in more detailed classification schemes. The Norwegian Olympic Committee (NOC) adopted a six-zone intensity model for elite endurance athletes (Tønnessen et al., 2024) and classifies Zones 1 and 2 (Z1, Z2) as low-intensity effort, Zone 3 (Z3) as moderate intensity, and Zones 4 to 6 (Z4–Z6) as high-intensity training (Table 3).

**TABLE 3:** Intensity scale for elite endurance athletes.

Scale 6-Zone 3-zone		Heart rate (%max)	VO_2_ (%max)	Blood lactate (mmol / L)	RPE_Borg_ (6–20)
1	LIT	60–72	50–65	<1.5	10–12
2	LIT	73–82	66–80	1.5–2.5	13–14
3	MIT	83–87	81–87	2.5–4.0	15–16
4	HIT	88–92	88–93	4.0–6.0	17–18
5	HIT	>93	94–100	6.0–10.0	18–19
6	HIT	NA	NA	>10	18–20

*VO*_*2*_, oxygen uptake; *RPE*, rating of perceived exertion (based on Borg’s 6–20 scale); *HIT*, high intensity training; *MIT*, moderate intensity training; *LIT*, low intensity training.

The eight-zone model was employed by the Chinese National Canoe Team (Yu et al., 2023) and defined L1 to L3 to enhance aerobic capacity, L4 to concentrate on mixed aerobic-anaerobic capacity, L5 and L6 to foster anaerobic endurance, and L7 and L8 to prioritize speed capacity (Table 4). These advancements collectively indicate that a more scientific and nuanced classification of training intensity aims to better address athletes’ unique requirements and enhance training results.

**TABLE 4:** Intensity level and standard of Chinese kayak team water training load.

Intensity zone	Paddle Frequency (times/minute)			Heart Rate (bpm)	Blood lactate (mmol/L)
	K1	K2	K4		
L1	<50	<50	<50	<110	<2
L2	55-75	65-85	75-95	140-160	2-4
L3	70-85	80-95	90-105	160-170	4-6
L4	80-95	90-105	100-115	170-180	5-8
L5	95-110	105-120	115-130	>180	7-10
L6	100-115	110-125	120-135	>180	8-15
L7	115-130	125-140	135-150	max	5-9
L8	>130	>140	>150	max	<7

K1 is a single, K2 is a double and K4 is a quad.

### 2.3 The emergence of training intensity distribution models

In conclusion, all models, regardless of the number of zones included in categorization, are essentially based on intrinsic physiological responses to dynamic variations in exercise intensity, particularly in terms of energy metabolism and gas exchange. The subsequent models additionally integrate psychological indicators. Essential physiological indicators are the principal criterion for establishing zone boundaries, including heart rate (HR), blood lactate concentration, ventilatory thresholds (VT), lactate thresholds (LT), and maximal lactate steady state (MLSS).

For instance, a comparison of the initial three-zone model (Z1–Z3) with the more current five-zone model (z0–z5) reveals that both are based on analogous physiological concepts (Faude et al., 2009; Rivera Köfler et al., 2025). The three-zone model highlights the transitional phases in metabolic reactions by classifying training intensity into low, moderate, and high zones based on the aerobic threshold (VT1/LT1) and the anaerobic threshold (VT2/LT2). This is enhanced by the five-zone model through the further splitting of the low- and high-intensity domains, yielding a more operationally accurate zoning framework. Although there are variations in granularity, the physiological underpinnings of the five-zone model and the three-zone model exhibit significant consistency—the former is essentially an extension and refinement of the latter. Both are relevant for the meticulous regulation of training programs and the precise management of training load distribution.

The three-zone concept has been successfully improved by the many training zone classification techniques described above. A three-zone classification was built through the unique “inflection points” identified in gas exchange and energy metabolism at the two ventilatory or lactate thresholds and is considered the most typical and commonly utilized framework for delineating intensity.

**FIGURE 3:**
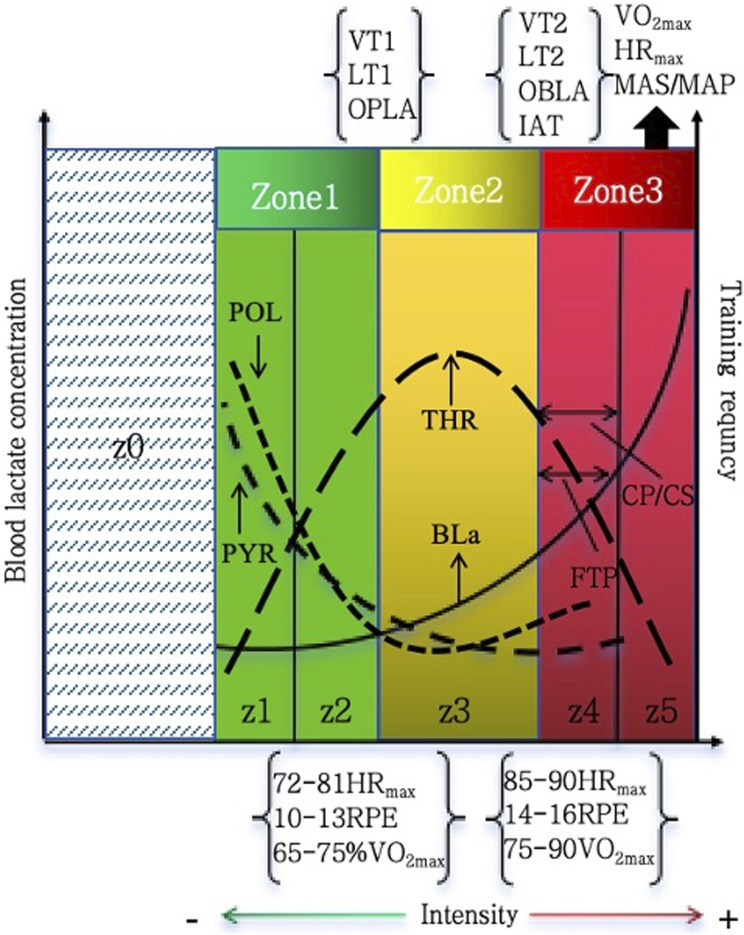
Comparative schematic of TID models and intensity zones. In this figure, threshold training is defined as the intensity between the first and second lactate turn points (LT1 and LT2) or near the maximal lactate steady state (MLSS). This corresponds to Zone 2 in the 3-zone model and Zone 3 in the 5-zone model. *Zone1*, training intensity zone 1 in the triphasic model; *Zone2*, training intensity zone 2 in the triphasic model;*Zone3*, Z3 in the triphasic model; *z0*,training intensity zone 0 in the 5-zone model; *z1*, training intensity zone 1 in the 5-zone model; *z2*,training intensity zone 2 in the 5-zone model; *z3*,training intensity zone 3 in the 5-zone model; *z4*,training intensity zone 4 in the 5-zone model;*z5*,training intensity zone 5 in the 5-zone model; *POL*, polarized training; *PYR*, pyramidal training; *THR*, threshold training;*BLa*,blood lactate;*VT1*,first ventilatory threshold; *OPLA*, onset of plasma lactate accumulation; *LT1*, first lactate threshold; *VT2*,second ventilatory threshold; *OBLA*, onset of blood lactate accumulation onset; *IAT*, Individual Anaerobic Threshold; *MLSS*, maximum lactate steady state; *LT2*, second lactate threshold; *VO*_*2max*_, maximum oxygen uptake; *HR*_*max*_, maximum heart rate; mas/map, maximum aerobic speed/maximum aerobic power; *vVO*_*2max*_, velocity associated with the maximum oxygen consumption; *CP/CS*, critical power/speed; *FTP*, functional threshold power; *RPE*,rating of perceived exertion in the Borg 6–20 scale.

Researchers, based on the framework, have progressively developed various specific Training Intensity Distribution (TID) models by measuring and allocating the training volume across intensity zones, combined with physiological parameters (Kong et al., 2024; Seiler and Kjerland, 2006), including the Polarized Training Model (POL), Lactate Threshold Training Model (THR), Pyramidal Training Model (PYR), and High-Intensity Training Model (HIT). The THR, PYR, and POL models have emerged as the three most prevalent, mechanistically defined, and empirically validated TID frameworks in contemporary endurance training research as competitive performance in athletes evolves and training science advances (Figure 3).

**FIGURE 4:**
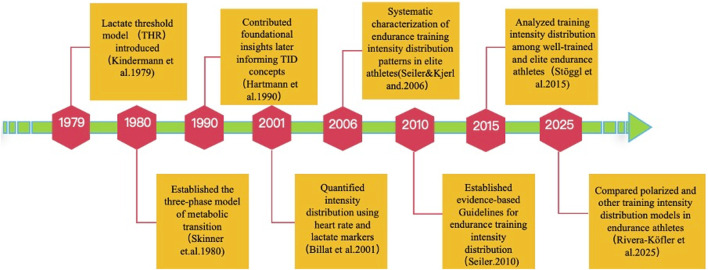
Evolution of Training Intensity Distribution (TID) Theory in Endurance Sports (1979–2025). POL, polarized training; *PYR*, pyramidal training; *THR*, threshold training.

The development of training intensity distribution (TID) has progressed over decades. The lactate threshold model (THR) was first introduced in the 1970s (Kindermann et al., 1979), followed by the three-phase metabolic transition model (Skinner and McLellean, 1980). Foundational insights that later informed TID concepts were provided (Hartmann et al., 1990), and intensity distribution was quantified using heart rate and lactate markers (Billat et al., 2001). Systematic characterization of TID patterns in elite athletes was reported (Seiler and Kjerland, 2006), and evidence-based guidelines were subsequently established (Seiler, 2010). Variations across performance levels were analyzed (Stöggl and Sperlich, 2015), and comparisons of polarized and other TID models demonstrated their effects on physiological outcomes and endurance performance (Rivera-Köfler et al., 2025). Figure 4​ contextualizes their historical progression.

## 3 Analysis of training characteristics, benefits, and mechanisms of the three main training modes

### 3.1 Pyramidal model (PYR)

#### 3.1.1 Analysis of training characteristics

**FIGURE 5:**
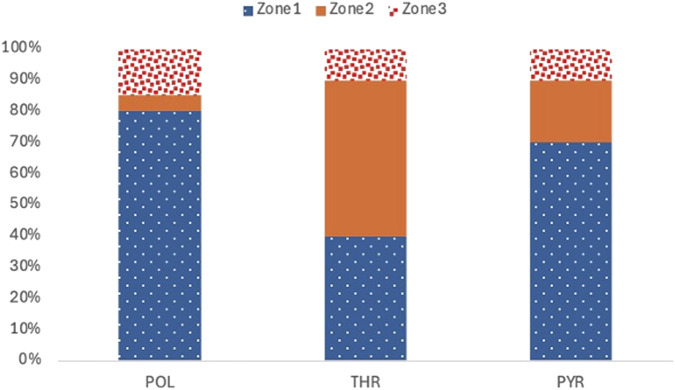
Training volume distribution chart for the three training models.

Coaches and researchers have been interested in the correlation between training intensity and volume to enhance the training outcomes of endurance events. The “pyramid” structural paradigm has been initially implemented in training practice since the 1950s The training load distribution across all levels of the pyramid model exhibits a negative correlation with exercise intensity. Specifically, low-intensity training should make up around 80% of the overall training volume, primarily focusing on low- and moderate-intensity intervals below the aerobic-anaerobic thresholds. Moderate-intensity training should not exceed 10% of the total training volume, while high-intensity anaerobic training should be confined to 5%–10% of the annual training volume (Sleamaker and Browning, 1996). Z1>Z2>Z3 (Figure 5) delineate the training distribution, with Z1 of relatively high proportion and accompanied by extended low-intensity training (Treff et al., 2019); Z1 and Z2 primarily utilize the aerobic oxidative system, whereas Z3 partly relies on the ATP-CP and anaerobic glycolysis systems. This model was based on aerobic metabolism and synchronized well with medium- and high-intensity training. It follows the fundamental principle of “negative correlation” between loading intensity and volume (i.e., training volume diminishes as intensity escalates). Consequently, it has received strong support from both theoretical and practical spheres, particularly in long-term endurance training regimens. The “pyramid” strategy is frequently thought to be the most effective method to achieve objectives in endurance training. The “pyramid” model is also esteemed, particularly in long-duration endurance training regimens.

#### 3.1.2 Physiological benefits and underlying mechanisms of training

The pyramidal training model mainly focuses on low-intensity training loads and intentionally combines moderate and high-intensity efforts, offering a theoretical framework for extensive adaptations across many energy systems in endurance training. Numerous empirical studies have examined how the model works to optimize energy metabolism and improve performance results within this framework. It is certified that the pyramidal training model can effectively stimulate multiple energy pathways and various muscle fiber types by combining a broad spectrum of training intensities (Stöggl and Sperlich, 2015; Kong et al., 2024). Zone 1 (Z1), Zone 2 (Z2), and Zone 3 (Z3) sessions are distributed evenly, which improves aerobic metabolism, glycolysis, and the phosphagen system, augments overall metabolic efficiency, and fosters structural and functional adaptations in both slow-twitch and fast-twitch muscle fibers.

Pyramidal training can comprehensively improve the physical functions of elite endurance athletes in performance. It augments the body’s regulatory capacity during exercise by primarily raising VO_2_max and improving running economy (Barstow and Molé, 1991). The prevalence of Low-intensity training is common because it fosters mitochondrial biogenesis and augments capillary density, both of which improves the aerobic energy supply capacity of peripheral tissues. Low-intensity exercise enhances mitochondrial content and function. It also increases metabolic enzyme activity by activating key molecular pathways such as AMPK and PGC-1α (Jørgensen et al., 2006; Baar et al., 2002; Handschin et al., 1989; Bassett and Howley, 2000). This provides crucial physiological support for recuperation and performance enhancement after high-intensity exercise.

A 2024 study reaffirmed the extensive advantages of pyramidal training for enhancing performance and improving body composition (Magalhães et al., 2024). After a 16-week intervention, amateur cyclists in the training group demonstrated a notable increase in lean body mass (from 76.16% ± 3.16%–78.11% ± 3.94%) and body water percentage (from 57.14% ± 3.50%–57.89% ± 3.97%) while showing a significant decrease in visceral fat (from 19.77% ± 3.41%–17.78% ± 4.19%) [27]. These findings corroborate the metabolic remodeling mechanisms of the pyramidal model, particularly the increasing of fatty acid oxidation through extensive Z1 training and the activation of glycolytic and anaerobic energy systems via limited Z3 sessions. They also highlight how widely applicable it is and how it can benefit the health of both elite and recreational endurance populations.

While these findings help explain performance gains, the concept of “durability” has also been proposed to account for fatigue resistance under prolonged low-intensity loads. This construct suggests that extensive Zone 1 training may enhance the ability to sustain performance despite accumulating fatigue. However, its empirical validation is still emerging. While most current explanations of durability remain theoretical—rooted in mechanisms like mitochondrial biogenesis and metabolic remodeling—preliminary evidence linking it to in-competition resilience has been documented (Maunder et al., 2021).

#### 3.1.3 Relative limitations

Even though the pyramidal model’s training advantages have been widely confirmed by several studies, its implementation in particular endurance disciplines or high-intensity, interval-centric sports remains comparatively constrained. This training approach may provide insufficient stimulus for high-intensity adaptations in elite long-distance runners. Suboptimal phosphagen system activation may result from the overemphasis on Zone 1 and Zone 2 training and the relatively short durations in Zone 3 (Casado et al., 2022). As the dominant energy source during brief, high-intensity efforts, this system primarily relies on the ATP–CP pathway and is characterized by rapid energy output over short durations. Deficient Z3 training may constrain athletes’ explosiveness and their recovery ability during high-intensity exertions. Furthermore, it will heighten tiredness and diminish training motivation and inferior training quality, adversely affecting total training efficacy. Consequently, although the pyramidal model is generally appropriate for endurance sports prioritizing aerobic capacity enhancement, its scant attention to high-intensity training may fall short of elite athletes’ requirements regarding explosive power, neuromuscular activation, and swift energy system adaptation (Cui et al., 2025). Training quality and competitive performance may be subsequently undermined as a result.

### 3.2 Lactate threshold model (THR)

#### 3.2.1 Analysis of training characteristics

In this review, threshold training (THR) is defined as exercise performed between the first and second lactate turn points (LT1 and LT2), or close to the maximal lactate steady state (MLSS). This corresponds to Zone 2 in the 3-zone model and Zone 3 in the 5-zone model, and this definition is applied consistently throughout the manuscript. The THR training model, first proposed in the late 1970s, defined the training zone as starting from the aerobic threshold (the blood lactate concentration first shows a marked rise) to the maximal lactate steady state (MLSS) or the anaerobic threshold (Kindermann et al., 1979). This model prioritizes continuous training at a moderate intensity, and its training zone is regarded as one of the most efficacious training zones due to the “golden intensity” of endurance training—the lactate threshold. Consequently, it became one of the first widely accepted models (Jones et al., 2019).

Moderate-intensity training that the THR model predominantly focuses on constitutes roughly 40%–60% of overall training duration, while low- and high-intensity represent approximately 30%–50% and 10%, respectively. The energy in this model mainly comes from aerobic oxidative metabolism, with little contribution from the ATP-CP system and anaerobic glycolysis.

The THR model primarily featured a substantial increase in training duration within the lactate threshold zone (Z2). This zone is sometimes referred to as “sweet spot training” in international literature, highlighting a balance between optimal metabolic efficiency and tolerable fatigue (Neal et al., 2013). The intensity distribution characteristic of THR training typically exhibits either a Z1 > Z2 > Z3 or Z2 > Z1 > Z3 pattern (Treff et al., 2019).

Prolonged training in this intensity zone can create a considerable physiological burden and possibly lead to severe fatigue (Seiler and Tønnessen, 2009). Therefore, it is necessary to arrange recuperation days or low-intensity sessions reasonably in the THR training to mitigate fatigue accumulation and improve training effectiveness.

Evidence supports THR effectiveness in several contexts, including swimming, triathlon, and middle-distance running, where technical efficiency is critical (Billat et al., 2001; González Ravé et al., 2021; Neal et al., 2013). These findings indicate that THR may be especially valuable when refining technique under sustained submaximal intensities.

#### 3.2.2 Physiological benefits and underlying mechanisms of training

The lactate threshold (THR) paradigm, which has long been regarded as a traditional and important strategy for improving endurance, places a strong emphasis on training inside the designated “golden intensity zone” of endurance training. Its training advantages and the physiological adaptation have garnered continuous scrutiny and have been fully corroborated for their impact on cardiovascular control and metabolic efficiency through extensive empirical research.

A 9-week continuous lactate threshold training can significantly improve heart rate economy, and a reported 2.7% ± 1.0% reduction in heart rate (P < 0.05) indicates favorable regulatory effects on the cardiovascular and autonomic nervous systems (Stöggl and Sperlich, 2014). Such a moderate-intensity continuous load is especially effective in increasing myocardial function and parasympathetic activity, which improves energy efficiency and running economy.

THR in elite endurance athletes is characterized by intensity distributions that emphasize low to moderate intensities, with high-intensity efforts making up only around 25% of the whole training duration (Rosenblat et al., 2019). This structure lessens the buildup of fatigue and facilitates sustained exercise performance over extended durations.

Low-to-moderate intensity training augments aerobic metabolism at the physiological level by elevating the activity of critical enzymes such as lactate dehydrogenase (LDH) and pyruvate dehydrogenase (PDH). This enables the lactate to transform into pyruvate and permits its entry into the tricarboxylic acid (TCA) cycle, thus augmenting aerobic metabolic efficiency and bolstering the body’s fatigue resistance (Brooks, 1986; Gudiksen et al., 2017). Furthermore, previous research suggests that elite athletes can enhance mitochondrial biogenesis and the expression of associated proteins through a training regimen that combines varied low-intensity training with a moderate amount of high-intensity sessions, leading to significant metabolic adaptations (Seiler and Kjerland, 2006).

Professional cyclists often display a pyramidal distribution when training load is measured by distance (Zapico et al., 2007). Similarly, distance runners and cross-country skiers frequently adopt PYR patterns during high-volume training phases, highlighting its role in sports with prolonged submaximal demands.

#### 3.2.3 Relative limitations

Similarly, there are certain limitations with the lactate threshold (THR) training model. Prolonged moderate-to-high intensity exercise associated with excessive activation of the sympathetic nervous system may cause hormonal exhaustion syndrome and markedly increase overtraining risk. Training-induced persistently elevated catecholamine levels are the main cause of this negative effect, which hinders the body’s ability to regulate physiological stress responses (Lucía et al., 2001). This training strategy is subject to more limitations in its application for adolescent athletes due to their underdeveloped neuromuscular and endocrine systems, which render them more vulnerable to training-induced stress. Moreover, excessive training loads can elevate the sports injury risk and negatively impact motivation and long-term athletic growth potential.

### 3.3 Polarized training model (POL)

#### 3.3.1 Analysis of training characteristics

The polarized training model developed in the early 21st century, allocates approximately 75% of training time to intensities below the lactate threshold, 15%–20% to high-intensity efforts well above the lactate threshold, and only about 5% (or none in some cases) to moderate-intensity training near the lactate threshold (Seiler and Kjerland, 2006). Based on the body’s major energy systems, this model conducts aerobic oxidation during low-intensity sessions and both glycolysis and the ATP–CP system during high-intensity efforts.

When the idea became popular, researchers encapsulated its principles into the renowned “80/20 Training Principle,” which states that 80% of training should be low-intensity activities (Fitzgerald, 2014) and the remaining 20% should consist of high-intensity work. The low-intensity component usually requires prolonged aerobic training to establish a solid aerobic foundation. Although the minimal level of moderate-intensity training attempts to mitigate the danger of overtraining, the high-intensity component concentrates on enhancing maximal oxygen absorption (VO_2max_).

The Polarization Index (PI), developed to quantify the degree of polarization within a training program, was in line with the evolution of the polarized training model (Treff et al., 2019). The first and second ventilatory thresholds (VT1 and VT2) are applied to divide training intensity into three zones—Z1, Z2, and Z3. The PI is calculated by the formula PI = log_10_ (Z_1_/Z_2_ × Z_3_ × 100), where Z_1_, Z_2_, and Z_3_ represent the percentage of total training time spent in Zones 1, 2, and 3, respectively. If PI is greater than 2, it indicates a polarized distribution; if below 2, it reflects a non-polarized structure. The modified formula PI = log_10_ [(Z_1_/(0.01 × Z_3_)) – (0.01 × 100)] is applied when Z_2_ equals zero. This framework indicates that the standard distribution pattern in polarized training is Z1 > Z3 > Z2 (Figure 5), with Zone 1 comprising the largest proportion and significantly outperforming Zones 2 and 3.

#### 3.3.2 Physiological benefits and underlying mechanisms of training

The polarized training model (POL) has received much attention in both theoretical studies and practical applications since its proposal. A significant number of researchers have assessed the efficacy of this paradigm in facilitating physiological adaptation and performance improvement in endurance athletes through systematic empirical investigations and comparative analyses. Many of them contrasted POL training with traditional approaches such as lactate threshold training and high-intensity interval training (HIIT) through controlled experimental methods. They furnish significant support for the scientific validity and practical usefulness of the POL method based on critical physiological metrics (such as VO_2_max, blood lactate responses, cardiopulmonary function) and performance indicators (like time-trial performance, running economy).

A 9-week intervention that involved 48 trained athletes (Table 5) demonstrated that polarized training produced the most increases in VO_2_max and peak blood lactate concentrations (Stöggl and Sperlich, 2014). Another study also received similar results that the significant enhancements in both 5 km performance and VO_2_max following a polarized training program further support the model’s effectiveness in promoting endurance adaptations and competitive performance gains (Neal et al., 2013).

**TABLE 5:** Changes in physiological indicators before and after the intervention.

	Polarized training		High-intensity interval training		Lactate threshold training	
	Pre	Post	Pre	Post	Pre	Post
VO _2peak_	60.6 ± 8.3	67.4 ± 7.7	63.7 ± 7.1	66.6 ± 5.8	63.2 ± 4.6	60.8 ± 7.1
[L-min-1-kg^-1^]		11.7% ± 8.4%		4.8% ± 5.6%		−4.1% ± 6.7%
HR_peak_ [bpm]	187 ± 7	186 ± 7	185 ± 9	182 ± 11	180 ± 10	179 ± 9
		−0.6% ± 1.9%		−1.3% ± 2.3%		−0.2% ± 1.9%
LA _peak_	10.2 ± 1.7	10.7 ± 1.7	9.6 ± 1.7	10.2 ± 1.7	9.5 ± 1.6	9.9 ± 2.2
[mmol-L^-1]^		7.5% ± 20.4%		6.4% ± 8.3%		5.3% ± 19.1%

*VO*_*2peak*_, peak oxygen uptake; *HR*_*peak*_, peak heart rate; *LA*_*peak*_: peak blood lactate.

Polarized training methodically integrates low- and high-intensity training elements to optimize both aerobic and anaerobic metabolic systems. Low-intensity exercise optimizes oxygen delivery and utilization by fostering mitochondrial biogenesis, elevating oxidase activity and the recruitment efficiency of slow-twitch muscle fibers, and improving capillary density and cardiac output. Meanwhile, high-intensity training involves the glycolytic and phosphagen systems, enhancing lactate clearance ability, lactate tolerance, and overall performance results (González Ravé et al., 2021). Low-intensity training is the foundation of the polarized model, which can significantly suppress sympathetic nervous system activity and enhance parasympathetic recovery capacity. This physiological equilibrium makes it easier to recover after a workout and lays a solid foundation for the effective execution of high-intensity training (Tønnessen et al., 2014).

Furthermore, research shows that POL training in Zone 3 can significantly increase VO_2_max and lactate metabolic capacity, primarily by triggering important molecular pathways like AMPK (AMP-activated protein kinase) and PGC-1α (peroxisome proliferator-activated receptor gamma coactivator 1-alpha). These mechanisms effectively promote mitochondrial biogenesis and foster oxidative metabolism (Jørgensen et al., 2006; Baar et al., 2002; Handschin et al., 1990).

Moreover, it has been demonstrated that polarized training significantly upregulates the expression of monocarboxylate transporters MCT1 by 10% and MCT4 in skeletal muscle by 133% (Neal et al., 2013). These changes improve lactate transport and oxidation efficiency, enhance energy metabolism pathways, increase the proportion of oxidative muscle fibers, and improve neuromuscular recruitment capacity, all of which add up to significant improvements in endurance performance.

#### 3.3.3 Relative limitations

The existing literature indicates that polarized training is not universally applicable to all people. High-intensity training, accounting for 20% of overall training volume for youth athletes, may exert undue stress on their developing neuromuscular and endocrine systems, thus obstructing their long-term athletic growth potential (Kindermann et al., 1979). Moreover, recreational athletes may be more prone to injury in polarized training owing to their constrained capacity to manage training loads (Chen, 2022; Carnes and Mahoney, 2019). Elite athletes with advanced training levels or greater capacity for adaptation might benefit from this training strategy. In addition, POL may not suit all populations. For youth and recreational athletes, the high-intensity load (∼20%) can increase neuromuscular stress and injury risk (Chen, 2022; Carnes and Mahoney, 2019). In technique-dependent sports such as rowing, reduced moderate-intensity training may limit skill acquisition. Over-reliance on POL in elite cohorts may also neglect the broader training spectrum needed for long-term athlete development.

## 4 Application characteristics of training periodization within the TID model

**FIGURE 6:**
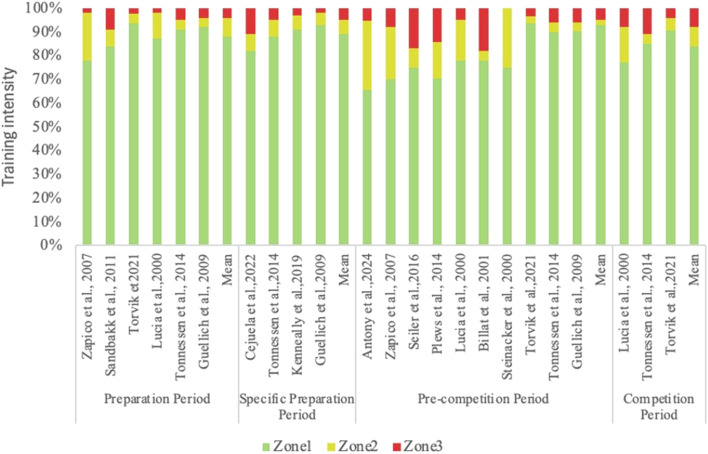
Training intensity distribution across different training phases in elite endurance athletes. Training intensity < VT1 or 2 mM in Zone1, ≤ VT2 or 4 mM in Zone2, and >4 mM in Zone3.

Training periodization theory’s evolution has established a definitive structural framework for managing training load in cyclic endurance sports, resulting in consistent and foreseeable patterns in training intensity distribution (TID) throughout several phases (Hu and Jin, 2020). ​ TID systematically shifts across macrocycles: (1) During general preparation, PYR/POL dominates (e.g., 78%–91% Zone 1 in rowing/cycling): to build aerobic foundations through mitochondrial biogenesis (Seiler and Tonnessen, 2009; Tonnessen et al., 2014); (2) Specific preparation increases Zone 3 focus (5%–10% in running, 15% in triathlon) while retaining PYR structure for sport-specific metabolic adaptation (Stoggl and Sperlich, 2015; Sperlich et al., 2023); (3) Pre-competition transitions to POL, with elite rowers reducing Zone 2 to <5% and elevating Zone 3%–15% to optimize competition readiness (Guellich et al., 2009).​ Within the periodized framework, distinct training activities, load distribution characteristics, and related energy system management methods result from the different objectives and physiological adaptation mechanisms inherent in each macrocycle phase (Figure 6; Table 6). As a result, the structural characteristics and theoretical foundations of TID throughout various training durations have thus been a primary topic of investigation in this domain.

**TABLE 6:** Training intensity distribution characteristics across training phases.

References	Sport	Intensity classification	Intensity zone	Intensity distribution		
Steinacker et al. (2000)	Rowing	Based on blood lactate	1.5 mM	75%		
			≥6.5 mM	25%		
Lucia et al. (2000)	Cycling	HR time-in-zone		Rest	Pre-comp	Comp
			<VT1	88%	78%	77%
			VT1-VT2	11%	17%	15%
			>VT2	2%	5%	8%
Billat et al. (2001)	Running	Training classified according to duration and velocity	>marathon pace	78%		
			= marathon pace	4%		
			<marathon pace	18%		
Billat et al. (2003)	Running	Training classified according to duration and velocity		LS	HS	
			>90min < vLT	83.8%	84.2%	
			= vLT	6.9%	14.4%	
			= v Δ 50%-vLT	4.3%	1.4%	
			= vVO2max	0%	0%	
Seiler et al. (2006)	Cross-country skiing	HR, sRPE, Blood lactate	Zone1:RPE≤4, ≤2mM, ≤VT1	∼75%		
			Zone2:RPE4-7,2–4 mM,VT1-VT2	5%–10%		
			Zone3:RPE≥7, ≥4mM, ≥VT2	15%–20%		
Zapico et al. (2007)	Cycling	HR time-in-zone		Winter	Spring	
			<VT1	78%	70%	
			VT1-VT2	20%	22%	
			>VT2	2%	8%	
Guellich et al. (2009)	Rowing	HR control based on lactate		1972:	2010:	
			<2 mM	40%	80%	
			2–4 mM	40%	12%	
			>4 mM	20%	12%	
Sandbakk et al. (2011)	Cross-country skiing	Session goal approach	Zone1: 1.5–2.5 mM, 60%–81%	Elite vs.	National	
			HRmax	84%	86%	
			Zone2:2.5–4mM, 82%–87% HRmax	7%	4.8%	
			Zone3:>4mM, >88% HRmax	8.7%	8.8%	
Tonnessen et al. (2014)	Cross-country skiing	Training time in lactate zones		1972:	2010:	
			<2 mM	40%	80%	
			2–4 mM	40%	2%	
			>4 mM	20%	12%	
Plews et al. (2014)	Rowing	Training time in lactate zones	<LT1	77.3%		
			LT1-LT2	16.9%		
			>LT2	5.8%		
Manunzio et al. (2016)	Cycling	Training time in lactate zones	<VT1	63%		
			VT1-VT2	28%		
			>VT2	9%		
Kenneally et al. (2020)	Running	Speed based on blood lactate	<LT1	91%		
			LT1-LT2	6%		
			>LT2	3%		
Torvik et al. (2021)	Cross-country skiing	Based on blood lactate		General	Pre-comp	Comp
			<2 mM	87%	88%	87%
			2–4 mM	4%	3%	5%
			>4 mM	0%	3%	4%
Cejuela et al. (2022)	Triathlon	blood lactate concentration	<LT1	82%		
			LT1-LT2	7%		
			>LT2	11%		
Stadnyk et al. (2024)	Cycling	Training time in lactate zones	<VT1	65.5%		
			VT1-VT2	29.2%		
			>VT2	5.3%		

*HR*, heart rate; *VT1*, first ventilatory threshold; *VT2*, second ventilatory threshold; *Prep*, preparation phase; *Comp*, competition phase; *Pre-Comp*, pre-competition phase; *LS*, low speed group; *HS*, high speed group; *LT*, lactate threshold; *vVO2max*, velocity at maximal oxygen uptake.

### 4.1 TID during the preparation phase

The general preparation phase initiates the macrocycle in cyclic endurance sports training to concentrate on enhancing aerobic metabolic capacity, increasing total training volume, and establishing fundamental physiological adaptations (Yu and Hu, 2019). This phase of endurance training seeks to improve mitochondrial density, cardiovascular function, and fat oxidation capacity to provide a firm metabolic and functional groundwork for future sport-specific advancement.

This phase is often defined by a High Volume, Low Intensity Training (HVLIT) plan, with Zone 1 seeing the majority of training intensities. Elite endurance athletes (e.g., in rowing, cycling, skiing) spend 78%–91% of training time in Zone 1 during preparation. Zones 2 and 3 comprise 2%–11% and 2%–9%, respectively (Seiler and Tønnessen, 2009; Tønnessen et al., 2014).

Zone 1 training is physiologically essential for improving the aerobic metabolic system. It augments the muscle’s capacity to use fatty acids by enhancing mitochondrial biogenesis and oxidative enzyme activity. In the meantime, it improves oxygen delivery and metabolite clearance by increasing capillary density and cardiac output. The parasympathetic nervous system is activated by this low-intensity, low-fatigue-cost training regimen, improving recovery potential and priming the body for forthcoming high-intensity demands (González Ravé et al., 2021).

Furthermore, the general preparation phase serves as a vital opportunity for technical education and movement reconfiguration. Zone 1 training’s constant and low-intensity characteristics enable athletes to enhance technical skills and efficiency with minimal fatigue, therefore boosting movement economy and sport-specific performance (Li, 2015).

The TID structure in this phase may differ depending on the training level. Although the deliberate incorporation of Zone 2 may enhance cardiopulmonary development, youth athletes predominantly depend on Zone 1 training (Stöggl and Sperlich, 2015; Yu et al., 2023). Conversely, elite athletes place a higher priority on accumulating the total training volume and concentrate on substantial amounts of low-intensity work to establish strong metabolic and neuromuscular foundations (Sleamaker and Browning, 1996).

Discipline-specific requirements also affect TID structure and training volume in the preparatory phase. This is prevalent in disciplines such as cycling and distance running with lower technical difficulty, extended training durations, and elevated Zone 1 proportions. Conversely, sports that prioritize movement coordination and integrated physical-technical skills, like rowing and biathlon, may have a higher percentage of moderate-intensity sport-specific sessions, which will facilitate simultaneous technical and physiological adaptation (Tønnessen et al., 2024; Guellich et al., 2009).

In this context, durability has been suggested as an underlying rationale for the heavy reliance on Zone 1 during preparation. The theory holds that accumulating large volumes of low-intensity work improves resistance to fatigue across long training or competition bouts. The current empirical foundation for durability remains limited, with its potential relevance to athletic performance acknowledged yet predominantly conceptual rather than empirically substantiated (Maunder et al., 2021).

### 4.2 TID during the specific preparation phase

A crucial stage in the training macrocycle, the specific preparation phase marks a vital transition from general conditioning to sport-specific performance. This phase aims to enhance sport-specific skills, cultivate endurance, and integrate high-intensity training. These objectives replicate competition demands and prepare athletes physiologically and psychologically for competition (Yu and Hu, 2019).

The core feature of this phase is the Zone 1 structure in Training Intensity Distribution (TID), while gradually augmenting the proportion of Zone 2 and Zone 3 training to simulate race intensity and pace requirements in actual competitions. Published data indicate that Zone 1 training constitutes around 70%–85% in conventional endurance sports like rowing, distance running, and cross-country skiing; Zone 2 accounts for 10%–20%; and Zone 3 increases to 5%–10% (Stöggl and Sperlich, 2015; Tønnessen et al., 2014; Sandbak et al., 2011). However, the percentage of Zone 3 may attain 11%–15% in interdisciplinary activities like triathlon (Sperlich et al., 2023).

The augmented load at moderate and high intensities in this phase physiologically enhances critical performance metrics like lactate clearance capacity, maximal oxygen uptake (VO_2_max), and neuromuscular recruitment efficiency. Zone 2 training can improve maximal lactate steady state (MLSS) and the efficiency of aerobic–anaerobic transition, whereas Zone 3 training offers more powerful stimulation to the cardiopulmonary system and neuromuscular coordination, which enhances competition-specific adaptation.

In addition, TID techniques vary throughout fields. Distance runners typically focus on Zone 3 stimuli to enhance pace-specific endurance (Billat et al., 2001), whereas cyclists frequently concentrate on high-volume Zone 1 training to sustain total training load (Zapico et al., 2007). The allocation of Zone 3 training should be judiciously restricted for youth athletes or people with diminished technical skill during this period to prevent undue fatigue and maintain movement quality.

### 4.3 TID during the pre-competition phase

As a pivotal step in the training macrocycle, the pre-competition phase signifies the shift from volume to quality and from general preparation to specificity. It mainly seeks to enhance and optimize sport-specific performance while maximizing athletes’ competitive preparedness through intensity management and training structure optimization.

Training Intensity Distribution (TID) varies greatly among sports during the pre-competition phase, contingent upon the discipline characteristics and the competition schedule. For example, elite rowers typically use a pyramidal TID during a 26-week pre-competition period: 77% Zone 1, 16% Zone 2, and 7% Zone 3 (Plews et al., 2014). This helps develop an aerobic base and enhances total training capacity. Rowers’ training intensity distribution (TID) significantly moves towards a polarized pattern as the competition approaches, especially in the final 6 weeks preceding the World Championships, with high-volume-low-intensity training (HVLIT) and high-intensity Zone 3 training constituting 90% to 95% of total training. The percentage of moderate-intensity training (Zone 2) drops significantly (Guellich et al., 2009). This transition attempts to mitigate fatigue risk by curtailing sympathetic nervous system overactivation, which was usually triggered via extended Zone 2 training. At the same time, high-intensity training facilitates more targeted conditioning by improving lactate clearance capacity and VO_2_max.

Training-induced adaptations during this period show distinct sport-specific differences between road cycling and distance running. Research demonstrates that cyclists generally maintain a pyramidal training pattern, with low-intensity training (Zone 1) accounting for the majority of training, whereas Zones 2 and 3 play a smaller role. In contrast, elite distance runners exhibit a TID pattern resembling polarization, with a substantial proportion of Zone 1 training, a notable decline in Zone 2, and an augmented focus on Zone 3. The structural disparities illustrate the unique energy system requirements and competitive traits of each sport: running competition has a shorter duration, and priority should be given to lactate tolerance and clearance, necessitating heightened high-intensity stimulation; cycling, on the other hand, emphasizes aerobic efficiency and total training volume, promoting low-intensity load accumulation to reduce fatigue (Tønnessen et al., 2024; Lucía et al., 2000).

Elite cross-country skiers and biathletes typically follow a pyramidal Training Intensity Distribution (TID) structure in the pre-competition phase, with Zone 1 constituting up to 92% of total training, while Zones 2 and 3 combined for only 8% (Seiler and Kjerland, 2006). This distribution prioritizes elevated training volume most economically and effectively to enhance aerobic capacity and technical consistency. The training intensity distribution (TID) pattern typically shifts towards polarization for top teenage cross-country skiers, with Zone 1 comprising around 75%, Zone 2 being between 5% and 10%, and Zone 3 increasing to 15%–20% (Seiler and Kjerland, 2006). This phase-dependent shift aligns with sport-specific energy demands.​​ While endurance-focused disciplines like cross-country skiing maintain high-Z1 pyramidal structures (92% Zone 1), explosive-power sports like distance running adopt polarized distributions to prioritize lactate tolerance. Crucially, evidence confirms that youth athletes require modified POL with higher Zone 3 (15%–20%) due to immature neuromuscular systems, whereas elite cohorts optimize through POL’s fatigue-reduction benefits (Seiler and Kjerland, 2006).

### 4.4 TID during the competition phase

During competition, TID stabilizes in a hybrid POL/PYR configuration to balance performance maintenance and recovery.​​ Empirical data reveals cyclists sustain 77% Zone 1 with Zone 3 up to 8% (Lucia et al., 2000), while cross-country skiers retain pyramidal distributions (88% Zone 1) even during intense racing schedules (Torvik et al., 2021). This reflects a strategic compromise: POL’s high-intensity elements address competition demands, while PYR’s low-intensity backbone facilitates inter-race recovery. The competition phase, which takes place right before competition, is a vital segment of the macrocycle. Its primary objectives are to sustain peak performance, ensure competitive preparation, mitigate fatigue risk, and facilitate swift recovery.

There is a relative lack of research data during the competition phase since Training Intensity Distribution (TID) is additionally affected by competition frequency, intensity, and recuperation needs. Current research suggests that most endurance athletes tend to implement a polarized training approach during the competitive season. The Training Intensity Distribution (TID) of elite cyclists shows that Zone 1 training accounts for about 77%, whereas Zones 2 and 3 comprise 15% and 8%, respectively. This structure is useful for the preservation of aerobic base fitness while also addressing the high-intensity requirements of competition (Lucía et al., 2000).

Analogous TID patterns are also seen in cross-country skiers and biathletes during competition, with augmented high-volume, low-intensity, and high-intensity training enabling athletes to sustain performance levels without inducing extreme fatigue.

The latest research indicates that the Training Intensity Distribution (TID) of top cross-country skiers may still maintain a pyramidal structure even during peak competition periods, with High Volume Low Intensity Training (HVLIT) comprising up to 88% of the training regimen (Torvik et al., 2021). This suggests that some athletes depend heavily on low-intensity training to facilitate recovery and physiological adaptation even amid repeated high-intensity racing.

Despite sport and individual variations, ​Zone 3 training generally increases slightly​ during competition periods. This underscores how crucial high-intensity capacity is in competition, and athletes need to make a significant modification in their training regimen as the season enters its final stage.

## 5 Investigation of the optimal training intensity distribution model: Consensus and controversies

The introduction of Training Intensity Distribution (TID) strategies, including the pyramidal model, lactate threshold model, and polarized model, in the middle of the 20th century has sparked continuous debate about which model improves endurance performance more universally and effectively in both academic and practical contexts. Researchers have come to recognize since the 21st century that the lactate threshold model, allocating most training to the moderate-to-high-intensity spectrum (approximately 60% of total training volume), may result in excessive training load and heighten the risk of fatigue accumulation and injury in the end (Li, 2015). Therefore, the polarized and pyramidal models, both of which prioritize low-intensity training complemented with minor amounts of high-intensity training, have increasingly acquired traction and shown favorable training results in empirical research (Stöggl and Sperlich, 2014; Kenneally et al., 2021; Sperlich et al., 2023).

### 5.1 Polarized training vs. lactate threshold training

The lactate threshold (THR) training concept is well known as a fundamental approach for improving endurance performance. Its fundamental idea is to enhance athletes’ performance at the lactate threshold via a substantial amount of moderate-to-high-intensity training. Subsequent research has revealed that this paradigm has various shortcomings, including concentrated training loads, elevated recovery demands, and an increased risk of fatigue and injury, especially when the total training volume is high (Seiler, 2010).

The polarized training approach, with a significant proportion of low-intensity training (about 75%–80%) and a small quantity of high-intensity activity (around 10%–15%), provides a more equitable load distribution. It efficiently mitigates recovery stress and the restricted adaptation efficiency usually associated with prolonged moderate-intensity training, thereby offering a more rational framework for balancing training outcomes and fatigue management.

The polarized training model has been related to significantly greater improvements in maximal oxygen uptake and maximal heart rate compared to threshold-based protocols (Stöggl and Sperlich, 2014). The time-trial performance, time to fatigue, and exercise economy under polarized training have also shown superior outcomes (Table 7) (Rosenblat et al., 2019).

**TABLE 7:** Meta-analysis results of time-trial performance and maximal oxygen uptake in endurance athletes.

Outcome	Study	Measurement	Group	Pre-intervention	Post-intervention	Within-group	Between-group
						Change ±SD	Difference (95% CI)
TT	Neal et al. (2013)	40 km cycle (min±SD)	POL (n = 11)	NA	NA	−2.36 ± 2.2	−1.9 (−2.4,-1.4)
			THR (n = 11)	NA	NA	−0.4 ± 2.9	
	Esteve-Lanao et al. (2007)	10.4 km run (min±SD)	POL (n = 6)	37.5 ± 2.1	34.9±NA	−2.6 ± 0.53	−0.6 (-0.74,-0.46)
			THR (n = 6)	37.9 ± 2.1	35.9±NA	−2.0 ± 0.29	
	Muñoz et al. (2014)	10 km run (min±SD)	POL (n = 15)	39.3 ± 4.9	37.3 ± 4.7	−2.0 ± 1.5	−0.6 (-0.78,-0.42)
			THR (n = 15)	39.4 ± 3.9	38.0 ± 4.4	−1.4 ± 1.2	
VO_2_max/peak	Stöggl et al. (2014)	VO_2peak_ (L·min^-1^±SD)	POL (n = 12)	4.4 ± 1.0	4.9 ± 1.1	0.5 ± 0.4	0.6 (0.19, 1.0)
			THR (n = 8)	4.4 ± 0.8	4.3 ± 9.2	−0.1 ± 3.3	
	Esteve-Lanao et al. (2017)	VO_2max_ (ml·kg^-1^min^-1^ ± SD)	POL (n = 6)	68.6 ± 5.9	NA	NA	NA
			THR (n = 6)	70.3 ± 9.7			
	Muñoz et al. (2014)	VO_2max_ (ml·kg^-1^min^-1^ ± SD)	POL (n = 15)	61.0 ± 8.4	NA	NA	NA
			THR (n = 15)	64.1 ± 7.3			

*SD*,standard deviation; *TT*, time trial; *POL*, polarized training; *THR*, threshold training; *NA*, not available; *VO*_*2max*_, maximal oxygen uptake; *VO*_*2peak*_, peak oxygen uptake.

In addition to enhancing energy system adaptability, polarized training reduces overtraining risk and allows athletes to achieve more consistent performance gains while maintaining high training volumes. This approach is especially advantageous for the progressive development of elite endurance athletes because it offers greater flexibility and long-term sustainability for coaches.

In conclusion, the polarized training model has gained wide attention in modern endurance training for its physiological and performance benefits. However, evidence also highlights the effectiveness of pyramidal and threshold models in certain contexts, underscoring that no single model is universally superior.

### 5.2 Polarized training vs. pyramidal training

The pyramidal and polarized training models have emerged as the predominant Training Intensity Distribution (TID) strategies among elite endurance athletes in recent years. Although both models present unique advantages in application, there is still some academic discourse concerning their comparative efficacy. Even though the pyramidal model is widely used because of its structural integrity and extensive applicability, training science advancements indicate an increasing trend towards transitioning from pyramidal to polarized structures, especially in cyclic endurance sports characterized by high training volumes (Table 8).

**TABLE 8:** Training load distribution in high-level endurance athletes (Guo et al., 2010).

Study	Sport	Intensity distribution			Intensity source
		LIT	MIT	HIT	
Orie et al. (2014)	Speed skating	75%–85%	10%–20%	5%–10%	Year-round training
Schumacher et al. (2002)	Cycling	94%	4%	2%	Year-round training
Esteve-Lanao et al. (2005)	Running	71%	21%	8%	Training from August to February
Billat et al. (2001)	Marathon	78%	4%	18%	All training before the Olympic test event
Billat et al. (2003)	Running	85%			All training 8 weeks before the race
Hartmann et al. (1989)	Rowing	70%–94%	5%–22%	1%–8%	Year-round water training
Zapico et al. (2007)	Cycling	70%–78%	20%–22%	2%–8%	All training from November to June
Sandbakk et al. (2011)	Cross-country skiing	80%–84%	5%–7%	9%	Endurance training 6 months before the test

*LIT*, low intensity training; *MIT*, moderate intensity training; *HIT*, high intensity training; LIT ≤ LT1,LT1 ≤ MIT ≤ LT2MLSS, HIT > LT2/MLSS.

Champions of the polarized training model contend that it offers substantial advantages in aerobic development, fatigue management, and training adaptation by combining a small portion of low-intensity training to strengthen the aerobic base with a small amount of high-intensity efforts to improve anaerobic metabolism and neuromuscular recruitment efficiency (Foster et al., 2022; Stöggl, 2018; Li et al., 2024; Li, 2013; van der Zwaard et al., 2021; Manzi et al., 2015).

Research indicates that this approach allows for longer, more effective training under equal loads while also diminishing the recovery demands and neural fatigue linked to moderate-intensity exertions. Additionally, polarized training’s high intensity efficiently engages type II muscle fibers, improving explosive strength and competition-specific performance, while also bolstering training motivation (Li, 2013).

**FIGURE 7:**
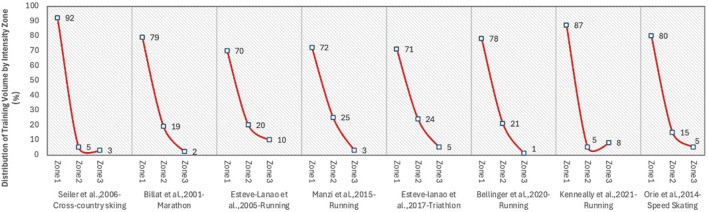
Time/distance intensity distribution in endurance athletes (Burnley et al., 2022)

Notwithstanding these findings, empirical training data reveal that the annual training regimen of numerous elite endurance athletes still follows a pyramidal distribution (Figure 7). The main cause of this disparity, according to Seiler et al., is the way that training load is measured: the distribution frequently appears polarized (with Zone 1 representing 75% and Zone 3 approximately 17%) when training sessions are assessed by frequency (like per session), but when evaluated by training time or distance, the distribution more closely resembles a pyramidal model, with Zone 1 constituting over 90% of total volume (Seiler and Kjerland, 2006). Some evidence indicates advantages of alternative models. Threshold-oriented training has been shown to be effective in swimming, where technical efficiency plays a key role (González-Ravé., 2021). Pyramidal distributions have also been observed in professional cycling when training volume is measured by distance (Zapico et al., 2007). Collectively, these findings suggest that PYR and THR may be preferable in certain disciplines or performance contexts.

**FIGURE 8:**
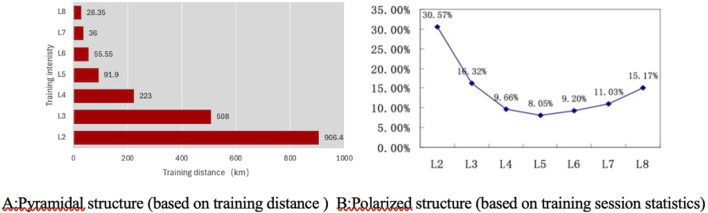
Training Intensity Distribution Structure of the Chinese Canoeing Team during the Olympic Preparation Year (Yu, 2015). **(A)** Pyramidal structure (based on training distance) **(B)** Polarized structure (based on training session statistics).

Empirical research on the Chinese national canoe team in an Olympic preparation year revealed that training intensity distribution became polarized as measured by session count (Figures 8–A) but appeared pyramidal when assessed by training distance (Figures 8–B). Polarized training provides universal benefits for elite endurance athletes, which has been questioned and led some researchers to argue that the current evidence is insufficient to support such a conclusion (Burnley et al., 2022). Comparative studies have demonstrated that certain elite athletes were able to achieve comparable or even superior outcomes by employing lactate threshold or pyramidal training models.

The results demonstrate that the selection of a training model should be flexible enough to accommodate the athlete’s unique attributes, training phase goals, and sport-specific requirements, rather than rigidly adhering to a purported “optimal model.”

Previous research indicates that the selection of the Training Intensity Distribution (TID) model is affected by several factors, and discrepancies in quantification methods are a major contributor to inconsistent model classification. The resulting training intensity distribution (TID) often exhibits a pyramidal structure when training load is assessed using the subjective rating of perceived exertion (RPE) scale. However, a more distinctly polarized distribution is typically seen in classifications based on heart rate zones (Ieno et al., 2021). There is now a great deal of variation in how TID is interpreted in real-world situations due to this disagreement.

The inherent attributes of specific sports also have a significant impact on the configuration of training loads, except for measuring requirements. A polarized paradigm that emphasizes extensive low-intensity training and few high-intensity sessions is more in accordance with disciplines that need significant aerobic metabolism, like cross-country skiing and marathon running. In contrast, activities like cycling, swimming, and rowing typically show a pyramidal distribution (Selles Pérez et al., 2019; Treff et al., 2017; Sperlich et al., 2023).

Training volume and periodization methodologies are significant influencing factors of intensity distribution. Seiler posits that athletes who engage in approximately 350 h every year frequently display a TID pattern akin to the lactate threshold model. Nevertheless, the Training Intensity Distribution (TID) typically transitions towards either polarized or pyramidal patterns when overall training volume surpasses 750 h annually (Seiler, 2016).

Empirical research from Chinese national teams has further validated that alterations in TID structure can result from different training phases. Female national canoe athletes in standard macrocycles generally adopt a polarized training intensity distribution pattern, characterized by a focus on aerobic capacity training. The ratio of anaerobic endurance and mixed energy system training escalates in microcycles, altering the load structure to a pyramidal configuration (Seiler and Tønnessen, 2009; Yu and Gao, 2020).

Recent research has increasingly focused on the TID models’ dynamic integration. A training sequence that began with a pyramidal model and ended with a polarized model produced the most substantial improvements in VO_2_max, lactate threshold velocities (vBLa2 and vBLa4), and 5 km time-trial performance, according to a study that involved 60 endurance runners and compared four intervention strategies. It is worth noting that the average performance in a 5 km race has reached 1.5%, a meaningful advancement for elite-level athletes (Filipas et al., 2022). This discovery suggests that deliberately altering TID structures across a training cycle could result in higher performance gains than strictly following a singular model.

In summary, there are specific application scenarios for both pyramidal and polarized training models, yet there is no clear-cut evidence of their comparative advantages. A more efficacious approach for elite endurance athletes may involve the dynamic modification and synthesis of both models, contingent on individual traits, training phases, and sport-specific requirements, to enhance long-term competitive progression. To facilitate the strategic selection and synthesis of these models, Table 9 offers a comparative analysis of their key features and practical considerations.

**TABLE 9:** Comparative analysis of TID models (PYR, THR, POL).

Model	Definition	Physiological targets	Strengths	Limitations	Sport-specific examples
Pyramidal (PYR)	Majority of training at low intensity (Z1), decreasing volume with rising intensity (Z1 > Z2 > Z3)	Broad adaptations across aerobic metabolism, glycolysis, and phosphagen system	Well-suited for high training volumes; supports technical work at low intensity; enhances VO_2_max and running economy	Limited high-intensity exposure may restrict neuromuscular and explosive adaptations in elite athletes	Cycling (distance-based volume), long-distance running, cross-country skiing
Threshold (THR)	Emphasis on training around lactate threshold (Z2, MLSS/VT2)	Improves lactate clearance, cardiovascular efficiency, and metabolic economy	Effective for raising performance at “golden intensity”; improves myocardial function and running economy	High fatigue risk; hormonal/excess stress if volume excessive; less suitable for youth athletes	Swimming; mid-distance running
Polarized (POL)	75%–80% low intensity (Z1), 15%–20% high intensity (Z3), minimal Z2	Enhances aerobic base (Z1) and anaerobic/neuromuscular adaptations (Z3)	Strong VO_2_max gains; reduces overtraining risk; efficient balance of adaptation and recovery	May be too demanding for youth/recreational athletes; less applicable for technical drills (e.g., rowing); long-term development needs caution	Distance running, cross-country skiing, triathlon

PYR:*Pyramidal*;THR:*Threshold*;POL:*Polarized;*Z1:*Zone 1*;Z2:*Zone 2*;Z3:*Zone 3*;*​*MLSS:*Maximal Lactate Steady State*;VT2:*Ventilatory Threshold 2*;VO_2_max:*Maximal Oxygen Uptake*.

## 6 Critical appraisal of methodological variability and future perspectives

The existing body of literature on Training Intensity Distribution (TID) has provided significant insights into the physiological adaptations associated with different training models. However, a critical appraisal reveals substantial methodological variability that complicates the direct comparison and synthesis of findings across studies. This section identifies these key sources of variability and proposes a framework for both future research and practical application to advance the field.

A primary source of methodological variability stems from the lack of a standardized approach for demarcating training intensity zones. Researchers frequently employ different physiological markers, such as lactate thresholds, ventilatory thresholds, and heart rate zones, often with varying protocols. For instance, the use of a fixed percentage of maximal heart rate versus heart rate reserve can lead to different interpretations of training load, while the specific determination of lactate thresholds (e.g., a fixed concentration like 4 mmol/L vs. an individualized lactate turn-point) introduces further discrepancies. This methodological variation makes it challenging to draw robust conclusions regarding the superiority of one TID model over another.

Furthermore, the diversity and characterization of athlete populations pose another significant challenge. Many studies incorporate a heterogeneous mix of participants, including highly trained elite athletes and recreational individuals. The physiological and adaptive responses to a given training stimulus can differ markedly between these groups, and without precise classification and reporting, the generalizability of findings is severely limited. Future research must place a greater emphasis on studying homogenous athlete cohorts to better understand the model’s effects on specific populations.

The use of different training load monitoring tools also contributes to this variability. The reliance on field-based heart rate data, often without direct physiological validation, can differ from laboratory-based gas exchange or lactate measurements. The lack of detailed reporting on the specific methods used for data collection and analysis hinders the reproducibility and validity of the research.

Based on these limitations, we propose the following directions for future research. First, adopting a standardized reporting framework, similar to the CONSORT statement for clinical trials, is essential. This would require authors to transparently report the specific criteria for intensity zone demarcation, the characteristics of the study population, and the precise methods for data collection. This would enhance the clarity and comparability of future studies. Second, there is a need for longitudinal research to examine how TID evolves over an athlete’s career and in response to different training phases.

Finally, bridging the gap between theory and practice is paramount. The practical application of TID models must be adaptable and individualized, not prescriptive.

## 7 Practical considerations for coaches and practitioners

Effective application of Training Intensity Distribution (TID) models requires consideration of multiple contextual factors rather than reliance on a single framework. Sport type plays an important role: running and cross-country skiing athletes often benefit from polarized training (POL) to maximize VO_2_max and lactate clearance, whereas professional cycling more frequently shows a pyramidal (PYR) distribution when training volume is measured by distance. In swimming and rowing, threshold (THR) or PYR structures are common, reflecting the importance of technical efficiency and sustained submaximal workloads. In triathlon, mixed distributions are frequently applied, with model selection varying according to the relative demands of each discipline and phase-specific goals.

Training phase further shapes model choice. During general preparation, PYR is advantageous for accumulating large volumes of low-intensity work and building aerobic capacity. In the specific preparation phase, coaches often introduce more Z2 and Z3 load, which may resemble a POL or hybrid distribution. As athletes approach competition, POL becomes more prevalent, reducing time spent in Z2 and emphasizing race-specific intensities. In the competition phase itself, a mixture of POL and PYR is commonly observed, balancing high performance with adequate recovery.

Athlete level is another determinant. Youth athletes should avoid excessive high-intensity exposure; PYR or moderate THR structures better support technical development and long-term progression. Recreational athletes, by contrast, are generally safer with PYR, occasionally supplemented with POL sessions to enhance aerobic fitness without excessive fatigue. Elite athletes, with greater recovery capacity, can tolerate POL or phase-specific transitions from PYR to POL.

Finally, individual physiological characteristics may guide distribution. Athletes with relatively low lactate thresholds may gain more from THR or PYR to improve performance at “golden intensity.” Those with high fatigue resistance or strong recovery ability may respond better to POL. In technique-dependent sports, where skill maintenance is critical, PYR or THR may be more effective than POL. These considerations highlight that coaches should align TID selection with the sport’s demands, training phase, athlete level, and physiological profile, rather than adhering to a single model.

## 8 Conclusion

Training Intensity Distribution (TID) has emerged as a critical element in sports training science, holding significant theoretical importance and practical applicability in cyclic endurance sports. While models such as Polarized (POL), Pyramidal (PYR), and Threshold (THR) each demonstrate value, the central finding of this review is that no single model is universally superior. We must recognize that each TID pattern possesses distinct structural characteristics and exhibits context-dependent advantages and limitations. Consequently, ideal TID selection should not be a rigid formula but rather a dynamic process. Model choice must be flexibly adjusted according to the sport’s nature, training phase, and individual athlete profile. For instance, POL often benefits endurance sports with high aerobic demands, PYR is common in high-volume training phases, and THR may be valuable in technically demanding contexts. Increasing evidence also suggests that hybrid or sequential applications (e.g., PYR followed by POL) may further optimize long-term development. Ultimately, flexibility and individualization are paramount for optimizing training effectiveness. At the same time, it should be noted that this review is narrative in nature with a structured search strategy; therefore, the conclusions presented here should be interpreted as an integrative synthesis rather than a definitive systematic evaluation.
