# Correction: Recent advances in training intensity distribution theory for cyclic endurance sports: theoretical foundations, model comparisons, and periodization characteristics

**DOI:** 10.3389/fphys.2026.1845248

**Published:** 2026-04-21

**Authors:** Qihao Sun, Yin Yu, Jiayue Cui, Simin Lin, Xiaohan Wang, Tian Zhou

**Affiliations:** 1 School of Sports Training, Wuhan Sports University, Wuhan, China; 2 Research Center for High-Quality Development of Characteristic Competitive Sports, Wuhan Sports University, Wuhan, China

**Keywords:** cyclic endurance sports, training model, training load, training intensity distribution, endurance training

The reference for Seiler and Tønnessen (2009) was erroneously written as:

“Seiler, S., and Tønnessen, E. (2009). Intervals, thresholds, and long slow distance: the role of intensity and duration in endurance training. *Sport Sci*. 13. doi: 10.1186/s40798-022-00438-7”

It should be:

“Seiler, S., and Tønnessen, E. (2009). Intervals, thresholds, and long slow distance: the role of intensity and duration in endurance training. *Sport Sci*. 13. Available online at: http://sportsci.org/2009/ss.htm”

The reference for Maunder et al. (2021) was erroneously written as:

“Maunder, E., Seiler, S., Mildenhall, M. J., Kilding, A. E., and Plews, D. J. (2021). The durability of endurance training: a systematic review and meta-analysis. *Sports Med.* 51 (8), 1611–1630. doi: 10.1007/s40279-021-01459-0”

It should be:

“Maunder, E., Seiler, S., Mildenhall, M. J., Kilding, A. E., and Plews, D. J. (2021). The importance of ‘durability’ in the physiological profiling of endurance athletes. *Sports Med*. 51 (8), 1619–1628. doi: 10.1007/s40279-021-01459-0”

The reference for Steinacker (2000) incorrectly included the DOI “10.1097/00005768-199807000-00022”. No DOI is assigned to this reference, and the DOI has therefore been removed.

The original article has been updated.

