# Supplementary material for: Recent advances in training intensity distribution theory for cyclic endurance sports: theoretical foundations, model comparisons, and periodization characteristics
Source: Front Physiol. 2025 Oct 15;16:1657892. doi: 10.3389/fphys.2025.1657892 (PMC12568352; doi:10.3389/fphys.2025.1657892)
Supplement: Supplementary file 1 [file Supplementaryfile1.docx]

Supplementary Material

# Supplementary Figures and Tables

## Supplementary Figures


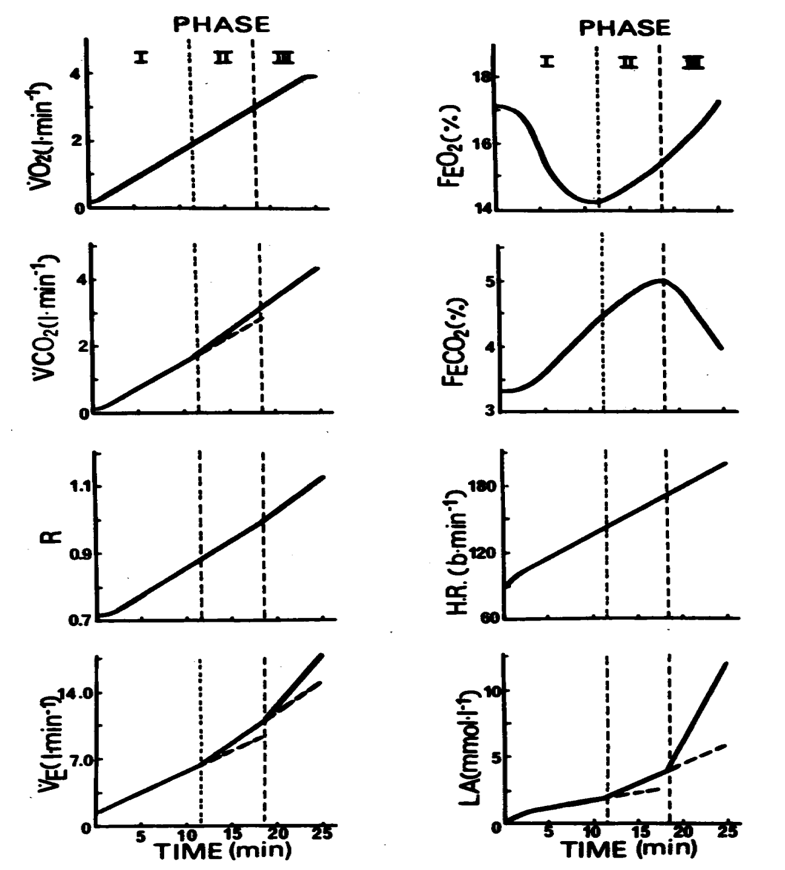


Figure 1 Hemodynamic representation of typical changes in blood lactate, heart rate, and selected gas exchange parameters during progressive exercise from rest to maximal oxygen consumption.( Skinner and McLellan,1980)

PHASE I - PHASE III refers to low to maximal intensity exercise. $\dot{V}$*O_2_*, oxygen uptake per minute;$\dot{V}$CO_2_, carbon dioxide expired per minute; *R*, respiratory quotient;$\dot{V}$*E*, pulmonary ventilation per minute; *FEO_2_*, expired oxygen concentration; *FECO_2_*, expired carbon dioxide concentration; *HR*, heart rate; *LA*, blood lactate concentration


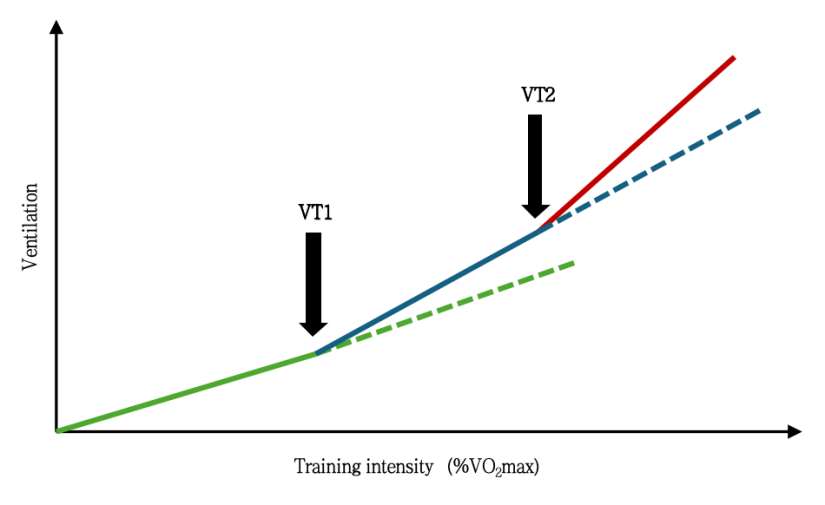


Figure 2 Ventilation Volume-Intensity Curve.


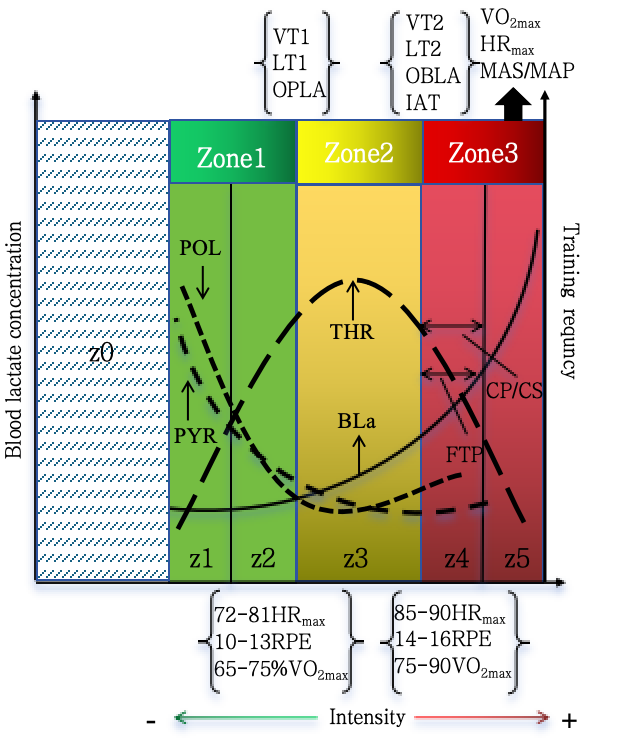
*VT1*, first ventilation threshold; *VT2*, second ventilation threshold.

Figure 3 Comparative Schematic of TID Models and Intensity Zones.

In this figure, threshold training is defined as the intensity between the first and second lactate turn points (LT1 and LT2) or near the maximal lactate steady state (MLSS). This corresponds to Zone 2 in the 3-zone model and Zone 3 in the 5-zone model. *Zone1*, training intensity zone 1 in the triphasic model; *Zone2*, training intensity zone 2 in the triphasic model；*Zone3*, Z3 in the triphasic model; *z0* ,training intensity zone 0 in the 5-zone model; *z1*, training intensity zone 1 in the 5-zone model; *z2*,training intensity zone 2 in the 5-zone model; *z3*,training intensity zone 3 in the 5-zone model; *z4*,training intensity zone 4 in the 5-zone model；*z5*,training intensity zone 5 in the 5-zone model; *POL*, polarized training; *PYR*, pyramidal training; *THR*, threshold training;*BLa*,blood lactate;*VT1*,first ventilatory threshold; *OPLA*,onset of plasma lactate accumulation; *LT1*, first lactate threshold; *VT2*,second ventilatory threshold; *OBLA*,onset of blood lactate accumulation onset; *IAT*, Individual Anaerobic Threshold; *MLSS*, Maximum Lactate Steady State; *LT2*, second lactate threshold; *VO_2max_*, maximum oxygen uptake; *HR_max_*,maximum heart rate; mas/map, maximum aerobic speed/maximum aerobic power; *vVO_2max_*, velocity associated with the maximum oxygen consumption; *CP/CS*, critical power/speed; *FTP*, functional threshold power; *RPE*,rating of perceived exertion in the Borg 6–20 scale.


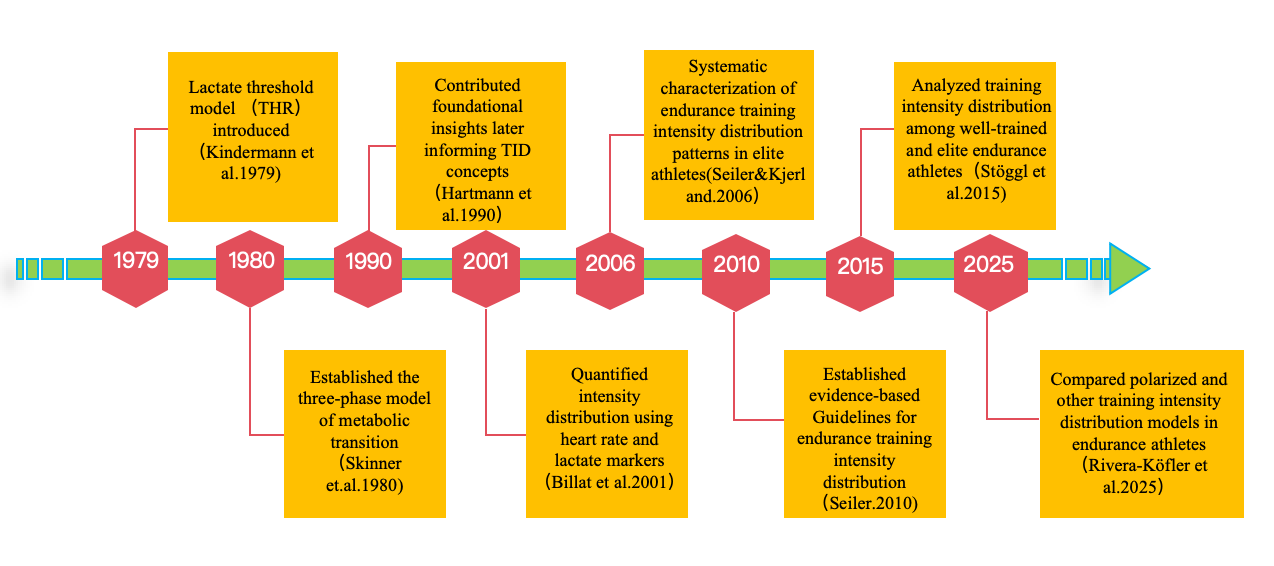


Figure 4 Evolution of Training Intensity Distribution (TID) Theory in Endurance Sports (1979–2025)​

*POL*, polarized training; *PYR*, pyramidal training; *THR*, threshold training


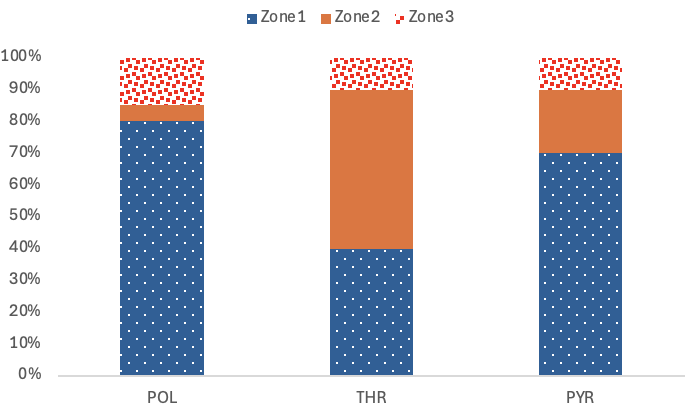


Figure 5 Training Volume Distribution Chart for the Three Training Models.


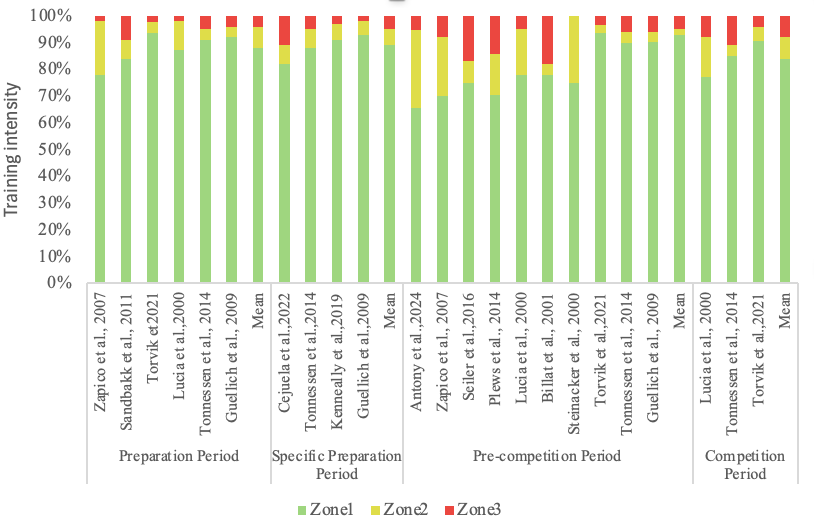


Figure 6 Training Intensity Distribution Across Different Training Phases in Elite Endurance Athletes.

Training intensity < VT1 or 2 mM in Zone1, ≤ VT2 or 4 mM in Zone2, and > 4 mM in Zone3.


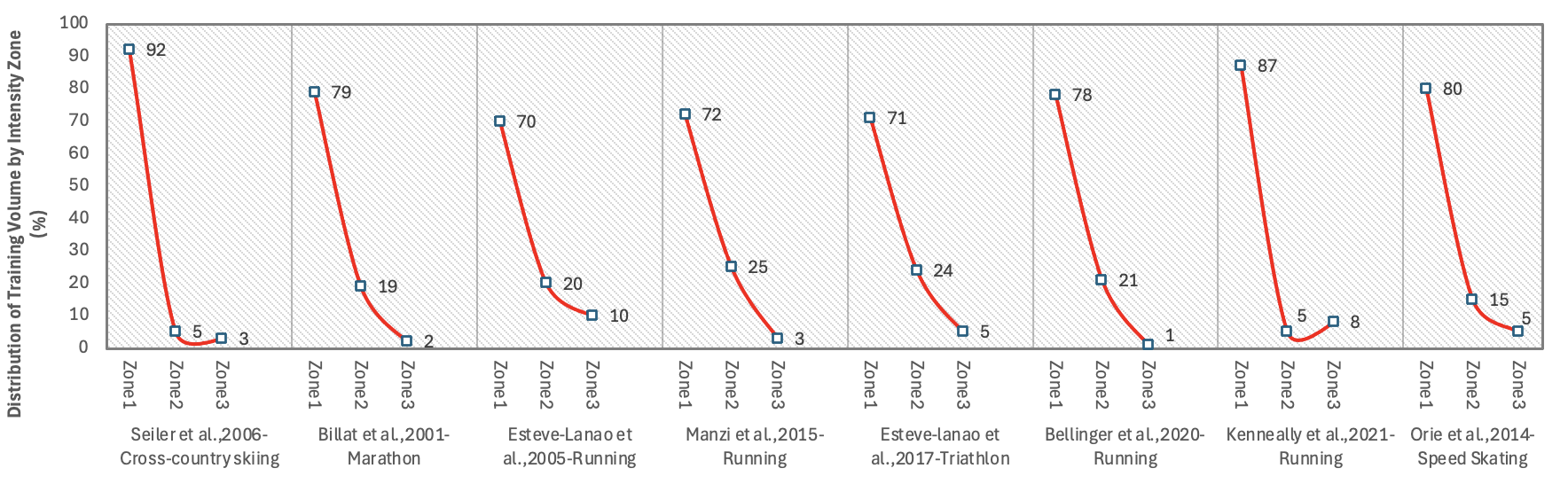


Figure 7 Time/Distance Intensity Distribution in Endurance Athletes (Burnley et al., 2022)


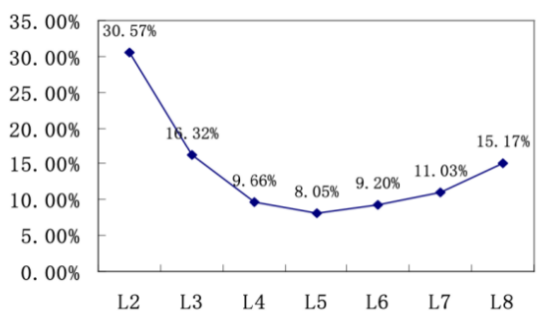

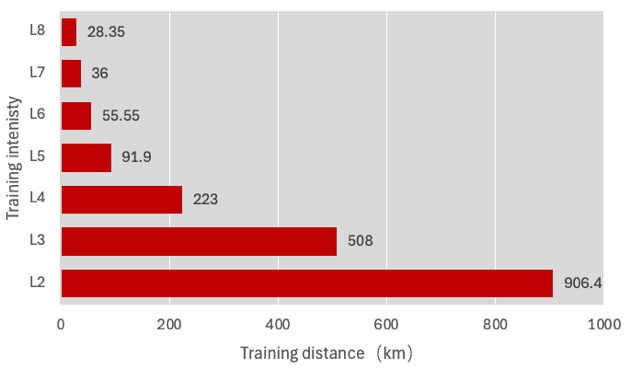


A:Pyramidal structure (based on training distance ) B:Polarized structure (based on training session statistics)

Figure 8 Training Intensity Distribution Structure of the Chinese Canoeing Team during the Olympic Preparation Year(Yu,2015)

## Supplementary Tables

Table 1 Summary of Key Characteristics of Included Studies

| Study | Sport | Sample Characteristics | Measurement Methods | TID Model | Key Findings |
| --- | --- | --- | --- | --- | --- |
| Kindermann et al.(1979) | Not mentiononed | n=20 | Blood lactate, ventilatory thresholds | THR (conceptual origin) | Foundation for THR model |
| Billat et al(2001) | Running (marathon) | Elite runners | HR, lactate, velocity | PYR | Typical PYR distribution observed; improved vVO₂max and running economy |
| Seiler et al.(2006) | Cross-country skiing | n = 12, world-class athletes | HR, RPE, lactate | POL | Introduced POL with 75–80% Z1, 5% Z2, 15–20% Z3 |
| Zapico et al.(2007) | Cycling | n=14 | HR, lactate | PYR | Distance-based training load showed PYR; session-based metrics suggested POL |
| Guellich et al.(2009) | Rowing | n=15 | HR, lactate | PYR → POL | Training shifted from PYR to POL before major competitions |
| Neal et al.(2013) | Cycling | n=22 | HR, lactate, MLSS | POL vs. THR | POL produced greater VO₂max and TT improvements |
| Stöggl et al.(2014) | Running & Cycling | n=48 | HR, VO₂, lactate | POL vs. THR vs. HIIT | POL showed superior VO₂max and performance gains compared with THR and HIIT |
| Pérez et al.(2020) | Ruuning | n = 20, ultra-endurance runners | VO₂max , fat metabolism analysis, MVC & RFD | POL vs THR | POL improved VO₂max and fat metabolism; THR better maintained neuromuscular function |
| Filipas et al.(2022) | Running (5km) | n=60 | HR, VO₂, TT performance | PYR → POL | \|  \| \| --- \|   Sequential PYR-to-POL led to greatest VO₂max and 5 km TT gains (+1.5%) |
| Sperlich et al.(2023) | Triathlon | n=30 | HR, VO₂, lactate | Mixed PYR / POL | Highlighted model integration depending on training phase and event demands |

*POL*, polarized training; *PYR*, pyramidal training; *THR*, threshold training;*HIIT*,high intensity interval training;*HR*,heart rate;RPE, rating of perceived exertion in the Borg 6–20 scale; *VO_2max_*, maximum oxygen uptake;*vVO_2max_*, velocity of maximum oxygen uptake; *MLSS*, maximum lactate steady state;*TT*,time trail；*MVC*, maximal voluntary contraction;*RFD*, rate of force development.

Table 2 Hypothetical Model of Various Thresholds and Phases Selected Characteristics during Progressive Exercise from Rest to Maximal Oxygen Consumption.( Skinner and McLellan,1980)

^
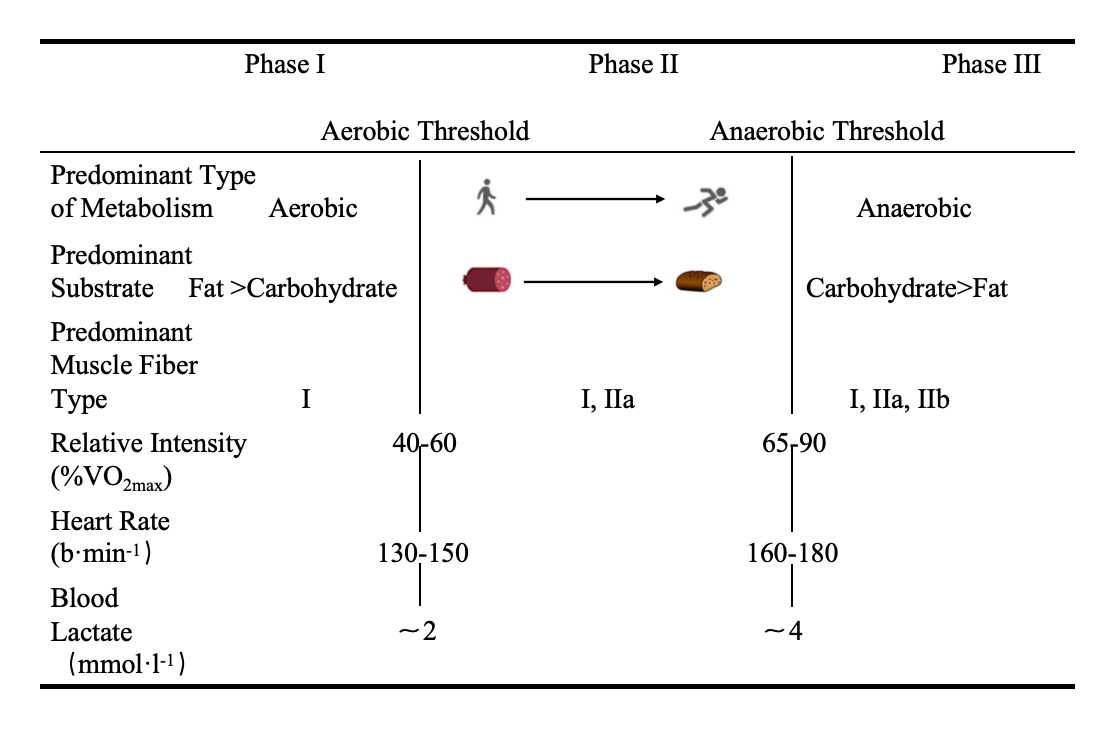
^

Table 3 Intensity Scale for Elite Endurance Athletes

| Scale  6-zone 3-zone | | Heart Rate  (%max) | VO_2_  (%max) | Blood Lactate  (mmol$/$L) | RPE_Borg_  (6-20) |
| --- | --- | --- | --- | --- | --- |
| 1 | LIT | 60-72 | 50-65 | <1.5 | 10-12 |
| 2 | LIT | 73-82 | 66-80 | 1.5-2.5 | 13-14 |
| 3 | MIT | 83-87 | 81-87 | 2.5-4.0 | 15-16 |
| 4 | HIT | 88-92 | 88-93 | 4.0-6.0 | 17-18 |
| 5 | HIT | >93 | 94-100 | 6.0-10.0 | 18-19 |
| 6 | HIT | NA | NA | >10 | 18-20 |

*VO_2_*, oxygen uptake; *RPE*, rating of perceived exertion (based on Borg’s 6–20 scale); *HIT*, high intensity training;*MIT*, moderate intensity training;*LIT*, low intensity training.

Table 4 Intensity Level and Standard of Chinese Kayak Team Water Training Load


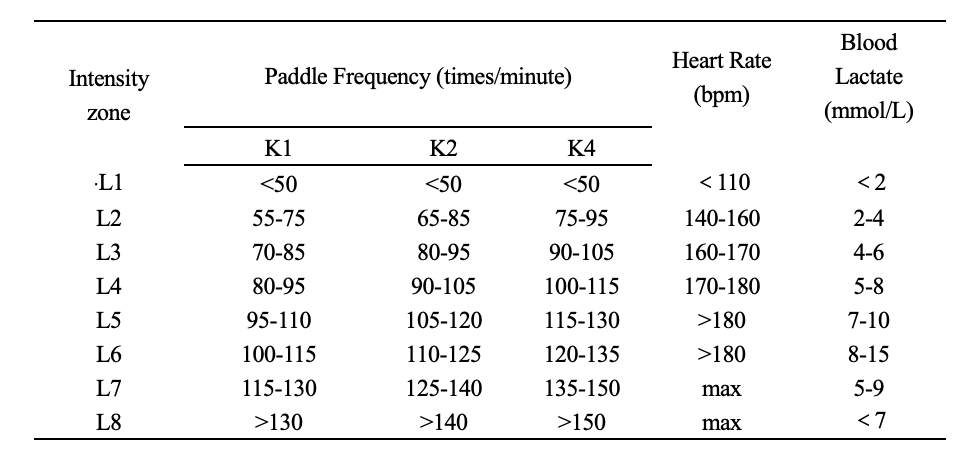


K1 is a single, K2 is a double and K4 is a quad.

Table 5 Changes in Physiological Indicators Before and After the Intervention

|  | Polarized Training | | High-intensity Interval Training | |  | Lactate Threshold Training | |
| --- | --- | --- | --- | --- | --- | --- | --- |
|  | Pre | Post | Pre | Post |  | Pre | Post |
| VO _2peak_  [L-min-1-kg^-1^] | 60.6±8.3 | 67.4±7.7  11.7±8.4% | 63.7±7.1 | 66.6±5.8  4.8±5.6% |  | 63.2±4.6 | 60.8±7.1  -4.1±6.7% |
| HR_peak_ [bpm] | 187±7 | 186±7  -0.6±1.9% | 185±9 | 182±11  -1.3±2.3% |  | 180±10 | 179±9  -0.2±1.9% |
| LA _peak_  [mmol-L^-1]^ | 10.2±1.7 | 10.7±1.7  7.5±20.4% | 9.6±1.7 | 10.2±1.7  6.4±8.3% |  | 9.5±1.6 | 9.9±2.2  5.3±19.1% |

*VO_2peak_*, peak oxygen uptake; *HR_peak_*, peak heart rate; *LA_peak_*: peak blood lactate.

Table 6 Training Intensity Distribution Characteristics Across Training Phases

| Reference | Sport | Intensity Classification | Intensity Zone | Intensity Distribution |
| --- | --- | --- | --- | --- |
| Steinacker et al. (2000) | Rowing | Based on blood lactate | 1.5mM  ≥6.5mM | 75%  25% |
| Lucia et al. (2000) | Cycling | HR time-in-zone | <VT1  VT1-VT2  >VT2 | Rest Pre-comp Comp.  88% 78% 77%  11% 17% 15%  2% 5% 8% |
| Billat et al. (2001) | Running | Training classified according  to duration and velocity | >marathon pace  =marathon pace  <marathon pace | 78%  4%  18% |
| Billat et al. (2003) | Running | Training classified according to duration and velocity | >90min <vLT  =vLT  =v$\Delta$ 50%-vLT  =vVO2max | LS HS  83.8% 84.2%  6.9% 14.4%  4.3% 1.4%  0% 0% |
| Seiler et al.  (2006) | Cross-country skiing | HR，sRPE，Blood lactate | Zone1:RPE≤4, ≤2mM, ≤VT1  Zone2:RPE4-7,2-4mM,VT1-VT2  Zone3:RPE≥7, ≥4mM, ≥VT2 | ∼75%  5-10%  15-20% |
| Zapico et al. (2007) | Cycling | HR time-in-zone | <VT1  VT1-VT2  >VT2 | Winter Spring  78% 70%  20% 22%  2% 8% |
| Guellich et al. (2009) | Rowing | HR control based on  lactate | <2mM  2-4mM  >4mM | 1972: 2010:  40% 80%  40% 12%  20% 12% |
| Sandbakk et al. (2011) | Cross-country skiing | Session goal approach | Zone1: 1.5-2.5 mM, 60-81%  HRmax  Zone2:2.5-4mM, 82-87% HRmax  Zone3 :>4mM, >88% HRmax | Elite vs. National  84% 86%  7% 4.8%  8.7% 8.8% |
| Tonnessen et al, (2014) | Cross-country skiing | Training time in lactate zones | <2mM  2-4mM   >4mM | 1972: 2010:  40% 80%  40% 2%  20% 12% |
| Plews et al.  (2014) | Rowing | Training time in lactate zones | <LT1  LT1-LT2  >LT2 | 77.3%  16.9%  5.8% |
| Manunzio et al. (2016) | Cycling | Training time in lactate zones | <VT1  VT1-VT2  >VT2 | 63%  28%  9% |
| Kenneally et al.  (2019) | Running | Speed based on blood lactate | <LT1  LT1-LT2  >LT2 | 91%  6%  3% |
| Torvik et al. (2021) | Cross-country skiing | Based on blood lactate | <2mM  2-4mM  >4mM | General Pre-comp Comp  87% 88% 87%  4% 3% 5%  0% 3% 4% |
| Cejuela et al. (2022) | Triathlon | blood lactate concentration | <LT1  LT1-LT2  >LT2 | 82%  7%  11% |
| Stadnyk et al. (2024) | Cycling | Training time in lactate zones | <VT1  VT1-VT2  >VT2 | 65.5%  29.2%  5.3% |

*HR*, heart rate; *VT1*, first ventilatory threshold; *VT2*, second ventilatory threshold; *Prep*, preparation phase; *Comp*, competition phase; *Pre-Comp*, pre-competition phase; *LS*,low speed group;*HS,*high speed group;*LT*, lactate threshold;*vVO2max*, velocity at maximal oxygen uptake.

Table 7 Meta-Analysis Results of Time-Trial Performance and Maximal Oxygen Uptake in Endurance Athletes

| Outcome | Study | Measurement | Group | Pre-intervention | Post-intervention | Within-group  change ± SD | Between-group  difference (95% CI) |
| --- | --- | --- | --- | --- | --- | --- | --- |
| TT | Neal et al.  (2013) | 40km cycle(min±SD) | POL (n=11)  THR (n=11) | NA  NA | NA  NA | -2.36±2.2  -0.4±2.9 | -1.9 (-2.4,-1.4) |
|  | Esteve-Lanao et al.  (2007) | 10.4 km run(min±SD) | POL (n=6)  THR (n=6) | 37.5±2.1  37.9±2.1 | 34.9±NA  35.9±NA | -2.6±0.53  -2.0±0.29 | -0.6(-0.74,-0.46) |
|  | Muñoz et al.  (2014) | 10km run(min±SD) | POL (n=15)  THR (n=15) | 39.3±4.9  39.4±3.9 | 37.3±4.7  38.0±4.4 | -2.0±1.5  -1.4±1.2 | -0.6(-0.78,-0.42) |
| VO_2_max/peak | Stöggl et al. (2014) | VO_2peak_(L·min^-1^±SD) | POL (n=12)  THR (n=8) | 4.4±1.0  4.4±0.8 | 4.9±1.1  4.3±9.2 | 0.5±0.4  -0.1±3.3 | 0.6 (0.19, 1.0) |
|  | Esteve-Lanao et al. (2007) | VO_2max_(ml·kg^-1^min^-1^ ± SD_)_ | POL (n=6)  THR (n=6) | 68.6±5.9  70.3±9.7 | NA | NA | NA |
|  | Muñoz et al. (2014) | VO_2max_(ml·kg^-1^min^-1^ ± SD_)_ | POL (n=15)  THR (n=15) | 61.0±8.4  64.1±7.3 | NA | NA | NA |

*SD* ,standard deviation; *TT* , time trial; *POL* , polarized training; *THR* , threshold training; *NA* , not available; *VO_2max_* , maximal oxygen uptake; *VO_2peak_* , peak oxygen uptake.

Table 8 Training Load Distribution in High-Level Endurance Athletes (Guo et al., 2010)

| Study | Sport | Intensity Distribution | | | Intensity Source |
| --- | --- | --- | --- | --- | --- |
|  |  | LIT | MIT | HIT |  |
| Orie et al.(2014) | Speed skating | 75%~85% | 10%~20% | 5%~10% | Year-round training |
| Schumacher et al.(2002) | Cycling | 94% | 4% | 2% | Year-round training |
| Esteve‑Lanao et al.(2005) | Running | 71% | 21% | 8% | Training from August to February |
| Billat et al.(2001) | Marathon | 78% | 4% | 18% | All training before the Olympic test event |
| Billat et al.(2003) | Running | 85% |  |  | All training 8 weeks before the race |
| Hartmann et al.(1989) | Rowing | 70%~94% | 5%~22% | 1%~8% | Year-round water training |
| Zapico et al.(2007) | Cycling | 70%~78% | 20%~22% | 2%~8% | All training from November to June |
| Sandbakk et al.(2011) | Cross-country skiing | 80%~84% | 5%~7% | 9% | Endurance training 6 months before the test |

*LIT*, low intensity training;*MIT*, moderate intensity training;*HIT*, high intensity training;LIT ≤ LT1,LT1 ≤ MIT≤ LT2MLSS, HIT> LT2/MLSS.

Table 9 Comparative Analysis of TID Models (PYR, THR, POL)

| Model | Definition | Physiological Targets | Strengths | Limitations | Sport-Specific Examples |
| --- | --- | --- | --- | --- | --- |
| Pyramidal (PYR) | Majority of training at low intensity (Z1), decreasing volume with rising intensity (Z1 > Z2 > Z3). | Broad adaptations across aerobic metabolism, glycolysis, and phosphagen system. | Well-suited for high training volumes; supports technical work at low intensity; enhances VO₂max and running economy. | Limited high-intensity exposure may restrict neuromuscular and explosive adaptations in elite athletes. | Cycling (distance-based volume), long-distance running, cross-country skiing. |
| Threshold (THR) | Emphasis on training around lactate threshold (Z2, MLSS/VT2). | Improves lactate clearance, cardiovascular efficiency, and metabolic economy. | Effective for raising performance at “golden intensity”; improves myocardial function and running economy. | High fatigue risk; hormonal/excess stress if volume excessive; less suitable for youth athletes. | Swimming ; mid-distance running. |
| Polarized (POL) | 75–80% low intensity (Z1), 15–20% high intensity (Z3), minimal Z2. | Enhances aerobic base (Z1) and anaerobic/neuromuscular adaptations (Z3). | Strong VO₂max gains; reduces overtraining risk; efficient balance of adaptation and recovery. | May be too demanding for youth/recreational athletes; less applicable for technical drills (e.g., rowing); long-term development needs caution. | Distance running, cross-country skiing, triathlon. |

PYR：*Pyramidal*；THR：*Threshold*；POL：*Polarized；*Z1：*Zone 1*；Z2：*Zone 2*；Z3：*Zone 3*；*​*MLSS：*Maximal Lactate Steady State*；VT2：*Ventilatory Threshold 2*；VO₂max：*Maximal Oxygen Uptake*.

PYR：*Pyramidal*；THR：*Threshold*；POL：*Polarized；*Z1：*Zone 1*；Z2：*Zone 2*；Z3：*Zone 3*；*​*MLSS：*Maximal Lactate Steady State*；VT2：*Ventilatory Threshold 2*；VO₂max：*Maximal Oxygen Uptake*.
